# Psychosocial and behavioral correlates of persistent pain post disease-modifying treatment change in rheumatoid arthritis: a 12-month cohort study

**DOI:** 10.1186/s42358-026-00540-4

**Published:** 2026-03-31

**Authors:** Lucy Zhao, Melissa Sweeney, Lewis Carpenter, Savia de Souza, Emma Caton, James Galloway, Andrew Cope, Kirsty Bannister, Elena Nikiphorou, Rona Moss-Morris, Sam Norton

**Affiliations:** 1 Wolfson Sensory, Pain, and Regeneration Centre, King’s College London, London, UK; 2 Centre for Rheumatic Diseases, King’s College London, London, UK; 3 Department of Psychology, King’s College London, London, UK; 4 University of Hertfordshire, Hatfield, UK; 5 Department of Life Sciences, Imperial College London, London, UK; 6 Centre for Education, Faculty of Life Sciences and Medicine, King’s College London, London, UK

**Keywords:** Persistent pain, Rheumatoid arthritis, Depression, Anxiety, Cognitive and behavioral response to symptoms

## Abstract

**Background:**

Pain is a debilitating and persistent symptom of rheumatoid arthritis (RA), associated with impaired functional capacity and reduced quality of life. Despite targeted disease-modifying treatments, many RA patients experience persistent pain, suggesting mechanisms operating independently of classic inflammatory pathways. Psychological factors, such as depression and anxiety, can impact patterns of appraisal and behavioral coping strategies. Understanding how these associations underpin pain symptoms in RA is key for developing targeted symptom management interventions. Using data from the Patient-Reported Outcomes in patients with Persistent Rheumatoid Arthritis (PROsPer-RA) cohort, pain trends were assessed over 12 months following disease-modifying adjustment, mapping these against socioeconomic, psychological, and behavioral factors to identify associations and mechanisms.

**Methods:**

This prospective study followed RA patients recruited to PROsPer-RA switching or escalating disease-modifying treatment. Clinical and patient-reported outcomes were collected at baseline, 3, and 12 months, including disease activity, joint pain (visual analog scale), widespread pain index (WPI), depression and anxiety symptoms, and cognitive and behavioral responses to symptoms. Latent growth curve models estimated associations between predictor variables and pain outcomes at baseline and over time. Mediation analyses examined whether cognitive and behavioral responses mediated the relationship between depression/anxiety symptoms and pain outcomes.

**Results:**

Among 209 eligible patients, baseline joint pain was 49.8$\:\pm\:$24.9 out of 100 (worst pain) and WPI was 4.9$\:\pm\:$3.6 out of 17 areas, which persisted at similar levels at 3 and 12 months. Higher baseline pain was associated with male gender, financial stress, lower education, and unemployment. Higher levels in both pain outcomes associated with worse depression and anxiety symptoms at baseline and over time. Avoidance-based behavioral responses, particularly fear avoidance, were associated with worse pain and mediated the relationship between depression/anxiety symptoms and both pain outcomes.

**Conclusions:**

In this RA cohort, pain symptoms persisted at moderate-to-high levels over 12 months despite changing disease-modifying treatments. These symptoms were driven by psychological and behavioral factors alongside socioeconomic factors. Current RA therapies may be insufficient without considering the non-inflammatory processes driving persistent pain. Disease management should incorporate strategies that target mental health as well as cognitive and behavioral response patterns to symptoms.

**Supplementary Information:**

The online version contains supplementary material available at 10.1186/s42358-026-00540-4.

## Introduction

Rheumatoid arthritis (RA) is a chronic autoimmune condition and the most prevalent form of inflammatory arthritis. It is characterized by painful swelling of the joints, which significantly impacts patient’s mobility and quality of life. Pain is reported as a top priority by affected patients [1], yet its management is often suboptimal [2]. Current disease-modifying anti-rheumatic drugs (DMARDs) are highly effective in suppressing immune activation and inflammation, however, despite this, more than half of patients still experience clinically significant levels of persistent pain [1, 3]. Alongside joint pain, as many as a third of RA patients experience widespread pain, where their pain extends to non-joint areas, thereby intensifying their pain symptoms [4–6].

The persistence and diffusion of pain despite effective anti-inflammatory therapies suggest the involvement of mechanisms beyond inflammatory processes, such as the significant psychological impact of the condition, which remains a critical unmet need for patients. Emerging evidence shows that the prevalence of depression in RA is at least twice as high as that of the general population, with approximately 20% meeting the diagnostic criteria for major depression disorder and, depending on the methods used, 20–40% experiencing high levels of depression and anxiety symptoms [7–10]. Not only is this burden present early on at diagnosis and likely to persist long-term, it is also associated with a worse prognosis and treatment response as well as work impairments and a reduced quality of life [11]. The relationship between psychological distress and pain in RA is complex and bidirectional. Distress can significantly impair patients’ ability to function and cope with their symptoms, leading to worse disease management and disability [12–14]. Without appropriate support and patient education, this can trigger a vicious cycle of poor coping strategies and cognitive appraisals, as well as worse symptoms and mental health, with each factor compounding the others and exacerbating the negative impact on overall functioning.

To develop effective pain management interventions, it is important to understand the mechanisms by which psychological and behavioral factors impact disease prognosis and treatment response. However, despite their recognized significance in RA, there remains a notable gap in the literature examining these factors in relation to pain-specific outcomes. Understanding this complex relationship is critical for identifying patients who would benefit from additional non-pharmacological interventions alongside DMARD treatment.

The purpose of this study was to address this issue by characterizing the prevalence and trajectories of persistent RA-pain and widespread pain symptoms over 12 months in RA patients who have recently changed their DMARD treatment or escalated their dose. We aimed to identify associated factors at baseline, including socioeconomic descriptors and psychological factors of depression and anxiety symptoms as well as symptom-related cognitive and behavioral responses. Additionally, we aimed to investigate whether these cognitive and coping patterns mediate the relationship between mental health and pain outcomes, providing insights into the potential effectiveness of coping-focused interventions for RA pain management.

## Methods

### Study population

PROsPer-RA is a longitudinal observational study of patients (aged $\:\ge\:$16 years) recruited from rheumatology clinics at UK-based hospitals between 01/2020 and 12/2021. Recruitment was paused 03–09/2020 due to COVID-19 pandemic. Eligible patients were invited to participate and complete follow-up visits, which took place at 3 and 12 months and included clinical assessments and a patient-reported outcome survey. Our inclusion criteria were confirmed RA diagnosis with recent change or dose escalation in current DMARD treatment. We excluded patients who had established disease (> 1 year on treatment), had a diagnosis of other inflammatory rheumatic disease or severe co-morbidities (e.g. mental health or respiratory issues), were unable to give written informed consent or participate effectively, and were unfit for study participation due to any medical or psychological conditions in the physician’s opinion. Our analysis included participants who had completed at least one patient-reported outcome (PRO) or clinical follow-up.

### Procedure and measures

Eligible patients were identified by treating clinicians during routine rheumatology appointments. A member of the clinical research team approached eligible patients, provided a written information sheet, and obtained written informed consent prior to any study procedures. During COVID-19 restrictions, consent was obtained by telephone or electronically, and clinical assessments were completed at the next feasible in-person visit. Baseline assessments were completed during the clinic visit and included a clinician-administered Disease Activity Score in 28 joints (DAS28) examination following local protocols. Questionnaires were self-completed by patients on paper or online using Qualtrics depending on preference. Follow-up assessments at 3 and 12 months were provided according to the initial preference. Ethical approval was granted by the London Queen Square Research Ethics Committee (Ref: 19/LO/1664).

### Socioeconomic descriptors

Socioeconomic descriptors of the patient cohort at baseline included age, gender, ethnicity, employment, and education level. Factors including body mass index (BMI) and financial insecurity were reported at baseline and follow-up visits. Age and body mass index (BMI) were included as continuous variables. Gender included patients reporting as male or female. Ethnicity subgroups were coded as patients reporting as White, Black, Asian, and other ethnicities, but were not distinguished in the analysis due to insufficient sample size per subgroup, thus ethnicity was analyzed as a binary variable indicating White and non-White patients. Level of education was coded as patients who had higher education (A-levels and above) or not. Employment indicated patients who were in work or not. Financial insecurity was included as a continuous variable, which was measured by questions about low household income, restricted fuel use, and financial strain (e.g. bill-paying difficulties).

### Clinical outcomes

Clinical measures of disease activity were collected at baseline visit, 3-month, and 12-month follow-ups. These included the widespread pain index (WPI) as well as disease activity score (DAS28) and its components of tender/swollen joint count (T/SJC), C-reactive protein (CRP) level, and patient global assessment (PtGA) of disease activity recorded on a 0-100 visual analogue scale (VAS) [15, 16]. A similar VAS for joint-related pain was also included. Other clinical outcomes included seropositivity, mental health treatment, steroid or opioid use, and comorbidities. The weighted total count of comorbidities was scored in accordance with the Rheumatic Disease Comorbidity Index (RDCI) [17], which included all 11 comorbidities.

### Psychological questionnaires

Depression and anxiety symptoms were assessed at all three time points using the 9-item Patient Health Questionnaire (PHQ9) and the 7-item Generalized Anxiety Disorder questionnaire (GAD7), both of which have been validated as a screening tool with good sensitivity and specificity for depression and anxiety, respectively [18]. PHQ9 asks about the frequency and severity of depressed mood, anhedonia, and suicide ideation over the past 2 weeks, whilst GAD7 asks the frequency and severity of anxious feelings and uncontrollable worrying over the past two weeks. PHQ9 and GAD7 scores range from 0 to 27 and 0–21 with higher scores indicating more severe depression and anxiety symptoms, respectively.

Symptom-related cognitive and behavioral responses were assessed using the 6-factor version of the 18-item Cognitive and Behavioral Responses to Symptoms Questionnaire (CBRQ) proposed by Ryan et al. (2018) which is a transdiagnostic measure of symptom interpretation and associated behaviors with good validity and reliability across several long-term conditions [19, 20]. This CBRQ version excludes the pain catastrophizing scale due to low loading factors and cross-loading issues identified in the study. It has two cognitive subscales, including damage beliefs and symptom focusing, as well as four subscales assessing behavioral responses to symptoms, including fear avoidance, embarrassment avoidance, resting avoidance, and all-or-nothing behavior.

### Statistics

Patient characteristics were summarized and tabulated based on demographic and clinical descriptors. Continuous data were presented as means and standard deviation (SD) and categorical data were presented by absolute numbers and percentages. Significance threshold was *p* < 0.05. The 0.5-SD change was used as a distributional estimate for calculating minimal clinically important differences (MCIDs) for WPI and pain VAS [21]. This method was selected due to the absence of validated anchor-based MCIDs reported for WPI in RA patients and was applied consistently to pain VAS for pragmatic reasons. Additionally, given our recruited RA cohort was clinically more selective than the general RA population, a distribution-based MCID approach, rather than applying external estimates, would more accurately reflect the variability representative of our study cohort.

All statistical analyses were performed and plotted in StataMP 18 (StatCorp LLC, Texas, USA). Descriptive statistics were obtained from complete cases.

### Latent growth curve models

We employed latent growth curve (LGC) models to examine trajectories of the dependent variables over time, including pain VAS ratings and WPI measured at baseline, 3 months, and 12 months. In these models, we specified intercept (I) and slope (S) as the two latent variables, with I representing baseline levels of pain symptoms and S representing the linear change in pain symptoms over time. The inclusion of a parametric S rather than a non-parametric S was determined using the goodness of fit measured by Bayesian Information Criterion (BIC) and Akaike Information Criterion (AIC). The loading for I was fixed at 1 across all time points, and the factor loadings for S were fixed at 0, 3, and 12, respective of the time points, to represent the unequal time intervals between measurement points. Both I and S were regressed on baseline variables, adjusting for age, gender, and ethnicity in all models. The residual covariance between I and S was not constrained.

We used the full information maximum likelihood with missing values (MLMV) estimator, which uses all available data while accounting for missing data. While the study experienced some missing data (approximately 20% at baseline), partly attributable to data collection challenges during the pandemic period, the MLMV approach effectively handles incomplete observations without requiring data exclusion. In cases where the above LGC models failed to converge, we estimated a fixed-effects LGC model instead, where the variances of the latent I and S were fixed to zero, therefore assuming all individual trajectories follow a single average growth curve.

### Mediation analysis

Using structural equation modeling (SEM), we performed mediation analyses to examine whether symptom-related cognitive and behavioral patterns mediate the association between baseline psychological distress and pain symptoms both cross-sectionally and over time. The mediation models tested the hypothesis that psychological distress influences pain outcomes through its effect on unhelpful cognitions and behaviors, consistent with theoretical frameworks suggesting that distressed individuals may adopt counterproductive coping strategies that subsequently exacerbate pain experiences. All mediation models adjusted for age, gender, and ethnicity. Statistical significance of indirect effects was assessed using bias-corrected bootstrap confidence intervals based on 1000 bootstrap samples. Model parameters were estimated using MLMV to handle missing data efficiently. We evaluated model fit using standard SEM fit indices, including the comparative fit index (CFI), Tucker-Lewis index (TLI), and root mean square error of approximation (RMSEA), with established cutoff criteria for acceptable model fit.

## Results

The analysis included a total of 209 patients with RA switching or escalating their DMARD treatment. The average age was 53.2 ($\:\pm\:$14.1) years, and most patients were female (73.2%) and of White ethnicity (67.0%). Key sociodemographics and clinical data are outlined in Tables 1 and 2. As shown in Table 2, on a scale from 0 to 100, pain VAS ratings reduced from 49.8 ($\:\pm\:$24.9) at baseline to 45.4 ($\:\pm\:$27.0) at 3-months, which was maintained at 12-months (45.9 $\:\pm\:$30.2), while, out of a total score of 17, WPI was reported on average 4.9 ($\:\pm\:$3.6) at baseline, which persisted at 3-months (4.2 $\:\pm\:$3.4) before rising to 5.1 ($\:\pm\:$4.3) at 12-months.

In this cohort, using a distribution-based (0.5-SD) approach, the MCID for pain VAS scores was 12.47 (i.e. a change of 12.5 points or more is considered clinically meaningful). The average reduction in pain VAS scores from baseline to 12-months was 4.0 points; 33 patients (15.8%) showed clinically meaningful improvement from baseline to 3 months, and 22 (10.5%) from baseline to 12 months. The MCID for WPI was 1.82 (i.e. a change of 2 points or more is considered clinically meaningful). The average reduction in WPI from baseline to 12-months was 4.0 points; 25 patients (12.0%) showed clinically meaningful improvement from baseline to 3 months, and 11 (5.3%) from baseline to 12 months. Based on the EULAR criteria for a good treatment response (DAS28 improvement $\:\ge\:$1.2), in this cohort, 37 (17.7%) patients showed good treatment response from baseline to 3 months and 26 (12.4%) showed good treatment response from baseline to 12 months.

### Pain symptoms and baseline sociodemographic descriptors

**Fig. 1:**
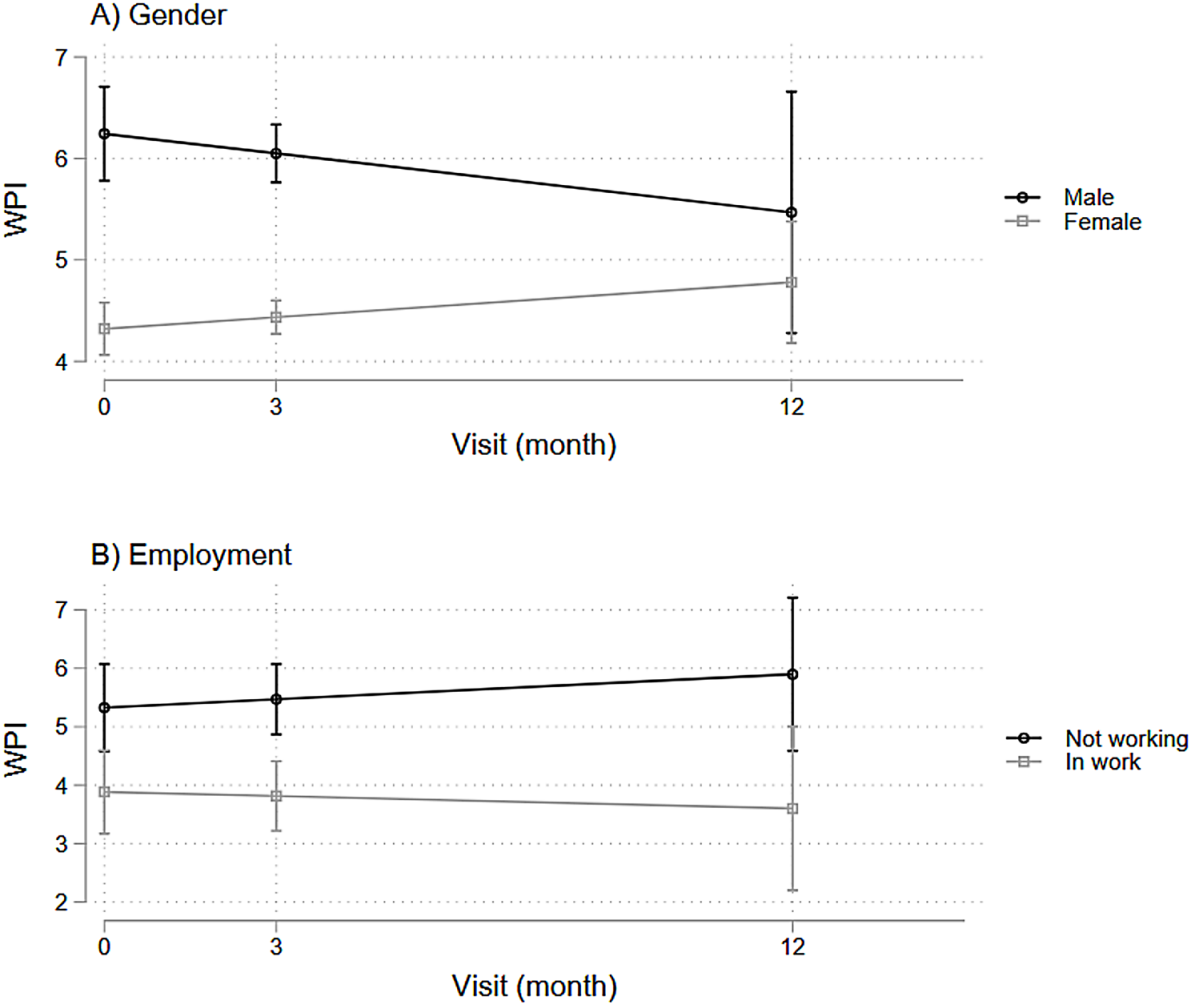
Associations between pain VAS and baseline sociodemographic and clinical descriptors

**Fig. 2:**
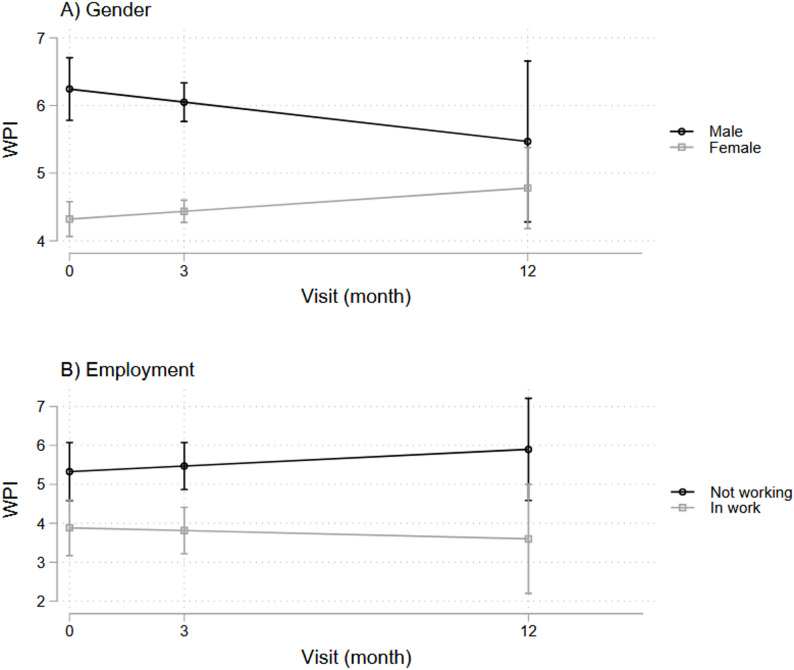
Associations between WPI and baseline sociodemographic and clinical descriptors

Using latent growth curve models (LGM), we examined both baseline associations and longitudinal trajectories for pain outcomes. For baseline pain levels (represented by the intercept in our LGM), we found several significant cross-sectional associations with sociodemographic factors, as shown in Figs. 1 and 2.

Higher pain VAS ratings were associated significantly with male gender (β-coefficient = 2.9, *p* = 0.034, 95%CI = [0.7, 18.2]), financial stress (β-coefficient = 5.2, *p* = 0.026, 95%CI = [0.6, 9.7]), and lower levels of education (β-coefficient = 10.8, *p* = 0.006, 95%CI = [3.1, 18.5]). Similarly, higher WPI was associated significantly with male gender (β-coefficient = 1.5, *p* = 0.031, 95%CI = [0.1, 2.9]), but was not significantly associated with financial stress nor lower education levels (see Supplementary tables S1, S2). Higher WPI was also significantly associated with unemployment (β-coefficient = 1.3, *p* = 0.035, 95%CI = [0.1, 2.5]).

In contrast to the baseline associations, none of these sociodemographic variables showed significant associations with pain VAS and WPI over time (see Supplementary Tables S1, S2).

**Table 1:** Study population demographics

Variable		
Patients, N (%)		209 (100)
Age, mean (SD)		53.2 (14.1)
Gender, N (%)	Male	56 (26.8)
	Female	153 (73.2)
Ethnicity, N (%)	White	140 (67.0)
	Black	8 (3.8)
	Asian	6 (2.9)
	Mixed	5 (2.4)
	Other	4 (1.9)
	Unknown	46 (22.0)
Prior depression, N (%)	Yes	21 (10.1)
	No	188 (89.9)
Financial security, N (%)	Secure	73 (34.9)
	Insecure	57 (27.3)
	Unknown	79 (37.8)
Higher education, N (%)	Yes	61 (29.2)
	No	101 (48.3)
	Unknown	47 (22.5)
Employment, N (%)	In work	75 (35.9)
	Not working	75 (35.9)
	Unknown	36 (17.2)
Seropositive (baseline), N (%)		138 (66.0)
Disease duration (years), mean (SD)		5.8 (7.5)
Ever used csDMARD^1^, N (%)	Yes	195 (98.5)
	No	3 (1.5)
	Unknown	11 (5.3)
On csDMARD^1^ at baseline, N (%)	Yes	173 (87.4)
	No	25 (12.6)
	Unknown	11 (5.3)
Ever used b/tsDMARD^2^, N (%)	Yes	45 (22.7)
	No	153 (77.3)
	Unknown	11 (5.3)
On b/tsDMARD^2^ at baseline, N (%)	Yes	37 (18.7)
	No	161 (81.3)
	Unknown	11 (5.3)
Baseline PHQ9, mean (SD)		8.6 (6.1)
Baseline GAD7, mean (SD)		5.4 (5.4)

^1^csDMARD=conventional synthetic DMARD

^2^b/tsDMARD=biologic/targeted synthetic DMARD

**Table 2:** Clinical outcomes at 0 months (baseline), 3 months, and 12 months

Visit (month)		Statistic	Pain VAS rating (0-100)	WPI	DAS28	TJC	SJC	CRP (mg/L)	PtGA (0-100)
0 (baseline)		Mean (SD)	49.8 (24.9)	4.9 (3.6)	4.2 (1.5)	6.1 (5.7)	3.8 (4.7)	11.1 (18.5)	54.4 (24.0)
		95%CI	[48.3, 51.2]	[4.7, 5.1]	[4.0, 4.4]	[5.2, 6.9]	[3.1, 4.5]	[8.4, 13.8]	[51.0, 57.7]
3		Mean (SD)	45.4 (27.0)	4.2 (3.4)	3.6 (1.6)	5.3 (6.1)	2.8 (3.8)	8.3 (15.6)	45.9 (25.0)
		95%CI	[43.4, 47.3]	[4.0, 4.5]	[3.3, 3.9]	[4.2, 6.3]	[2.2, 3.4]	[5.8, 10.8]	[41.1, 50.6]
12		Mean (SD)	45.9 (30.2)	5.1 (4.3)	3.3 (1.6)	4.0 (5.3)	2.3 (3.8)	7.5 (13.9)	44.4 (29.2)
		95%CI	[43.1, 48.6]	[4.7, 5.5]	[3.0, 3.6]	[3.0, 4.9]	[1.6, 2.9]	[5.1, 9.9]	[38.5, 50.3]

### Pain symptoms and baseline DAS28 composite score and components

**Fig. 3:**
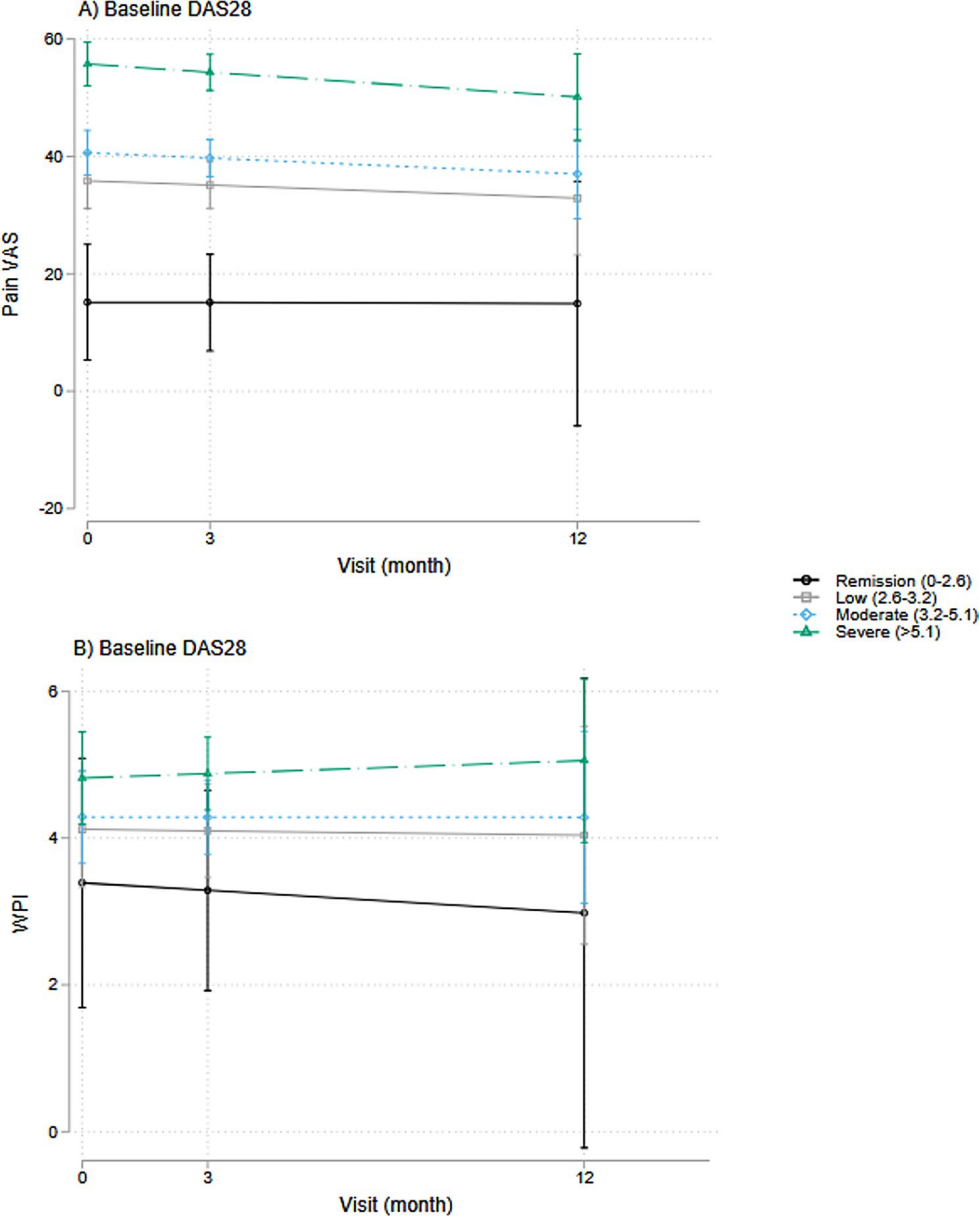
Associations between pain symptoms (pain VAS and WPI) and baseline DAS28 over time

Using LGM, we examined associations of baseline disease activity descriptors with pain outcomes at baseline (represented by the intercept of our LGM models) and over time (represented by the slope of our LGM models) and found several significant associations, as shown in Fig. 3.

At baseline, higher baseline DAS28 scores were significantly associated with higher pain VAS ratings (β-coefficient = 8.0, *p* < 0.001, 95%CI = [5.7, 10.2]) but not with WPI (Fig. 3). Pain VAS showed similar associations with DAS28 components, including TJC (sqrt) (β-coefficient = 9.6, *p* < 0.001, 95%CI = [7.2,12.0]), SJC (sqrt) (β-coefficient = 6.6, *p* < 0.001, 95%CI = [3.7,9.6]), and CRP levels (log-transformed) (β-coefficient = 4.37, *p* = 0.003, 95%CI = [1.5,7.3]).

Our longitudinal analysis showed that higher baseline DAS28 scores associated with persistent pain over time, including pain VAS ratings (β-coefficient = 7.2, *p* < 0.001, 95%CI = [4.4, 9.9]) and WPI (β-coefficient = 0.45, *p* = 0.029, 95%CI = [0.05, 0.85]) (Fig. 3). Specifically, TJC (sqrt) (β-coefficient = 2.0, *p* < 0.001, 95%CI = [1.3, 2.7]) and CRP (log-transformed) (β-coefficient = 3.6, *p* = 0.025, 95%CI = [0.5, 6.8]) were positively associated with higher pain VAS scores over time; whereas higher WPI was significantly associated only with TJC (sqrt) (β-coefficient = 0.6, *p* = 0.008, 95%CI = [0.2, 1.1]).

**Fig. 4:**
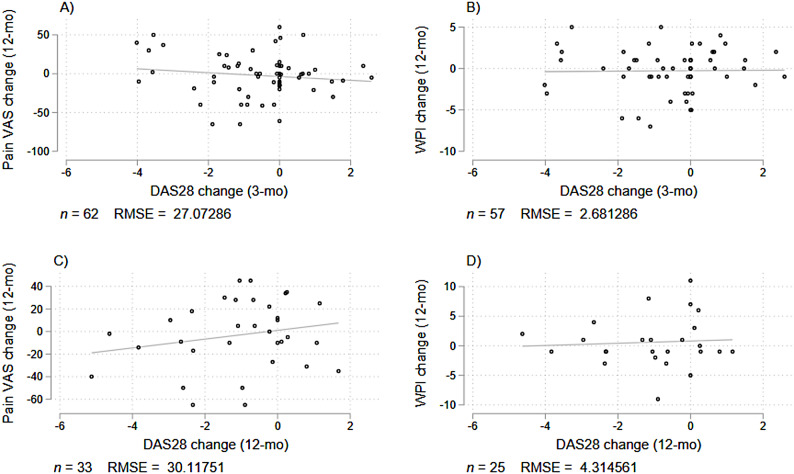
Correlation between changes in DAS28 scores and pain symptoms over time. During the 0–3 month period, DAS28 accounted for 1.7% and 0.02% of the variance in pain VAS and WPI (R^2^), respectively; during the 0–12 month period, DAS28 accounted for 4.3% and 0.4% of the variance in pain VAS and WPI (R^2^), respectively

Additionally, we examined whether DAS28 was the main contributing factor to the overall change in pain symptoms by performing a simple linear regression between DAS28 changes and pain VAS scores and WPI. As shown in Fig. 4, during the 0–3 month period, DAS28 accounted for 1.7% and 0.02% of the variance in pain VAS and WPI (R^2^), respectively; during the 0–12 month period, DAS28 accounted for 4.3% and 0.4% of the variance in pain VAS and WPI, respectively.

### Pain symptoms and baseline PHQ9 and GAD7

**Fig. 5:**
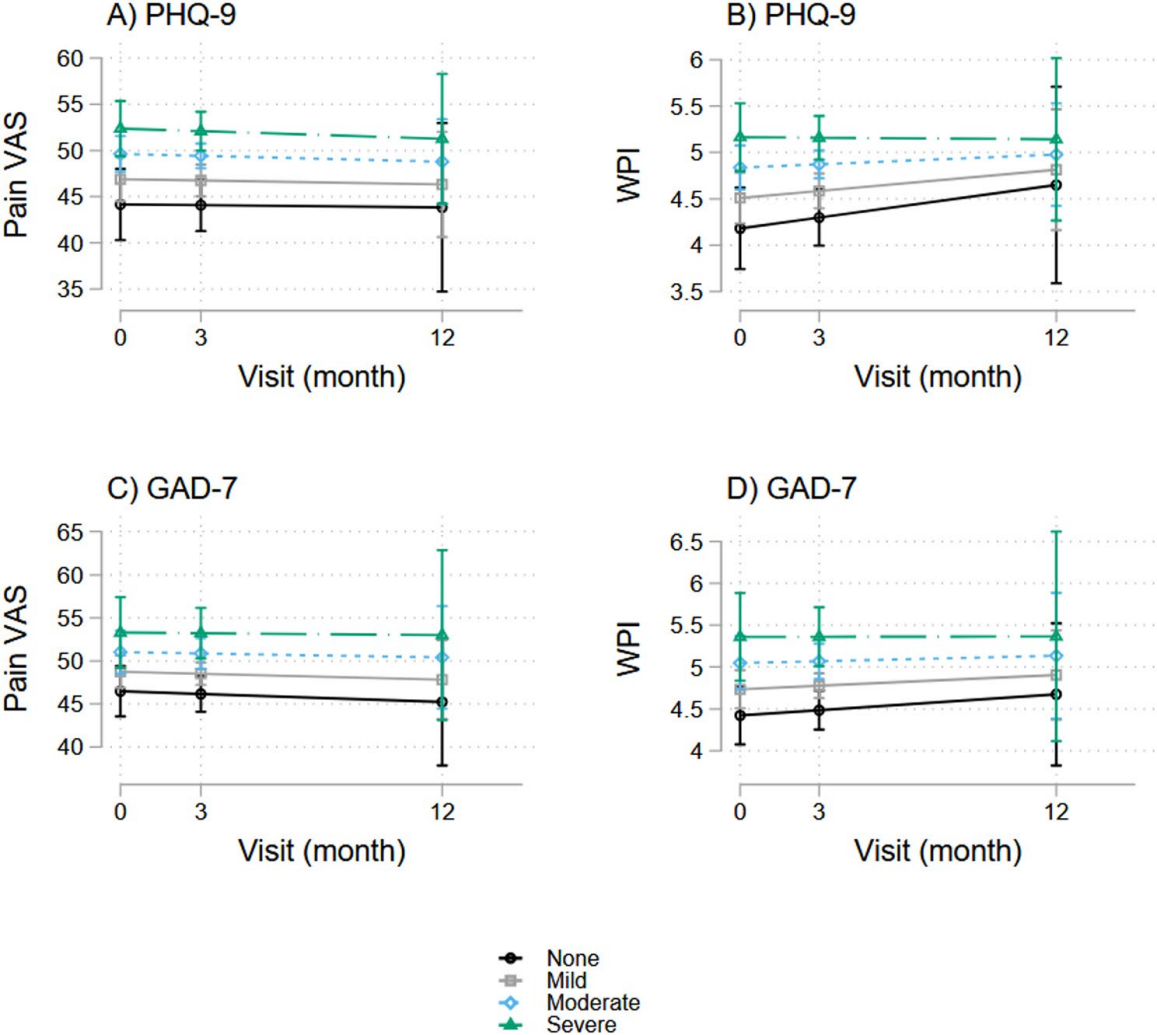
Associations between pain symptoms and baseline depression and anxiety symptoms

With our LGC models, we also examined baseline and longitudinal associations of both outcome variables of pain levels with patient-reported measures of psychological distress, which included PHQ8 and GAD7 screeners, and found significant associations at baseline and over time, as shown in Fig. 5.

At baseline, higher pain VAS scores associated significantly with higher scores on the PHQ9 depression screener (β-coefficient = 1.3, *p* < 0.001, 95%CI = [0.7, 1.9]) and GAD7 anxiety screener (β-coefficient = 1.2, *p* < 0.001, 95%CI = [0.5, 1.8]). A positive association was also observed between WPI and PHQ9 (β-coefficient = 0.2, *p* < 0.001, 95%CI = [0.1, 0.3]) and GAD7 (β-coefficient = 0.2, *p* < 0.001, 95%CI = [0.1, 0.3]) scores.

These associations were also reflected over time, as higher pain VAS scores associated with higher PHQ9 (β-coefficient = 1.4, *p* < 0.001, 95%CI = [0.7, 2.0]) and GAD7 (β-coefficient = 1.4, *p* < 0.001, 95%CI = [0.6, 2.2]) scores, whereas higher WPI associated with higher PHQ9 (β-coefficient = 0.2, *p* < 0.001, 95%CI = [0.2, 0.3]) and GAD7 (β-coefficient = 0.3, *p* < 0.001, 95%CI = [0.2, 0.4]) scores.

### Pain symptoms and cognitive and behavioral responses

**Fig. 6:**
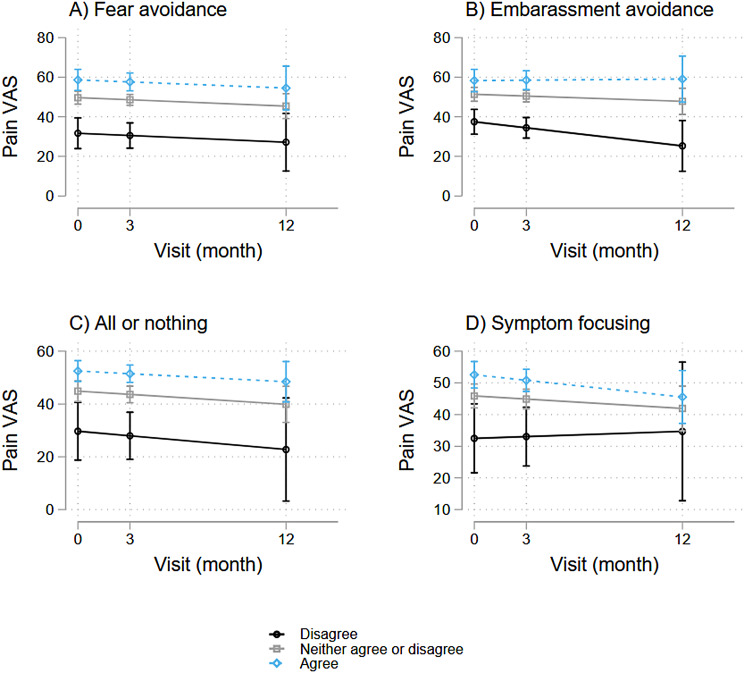
Associations between pain VAS and baseline cognitive and coping strategies

**Fig. 7:**
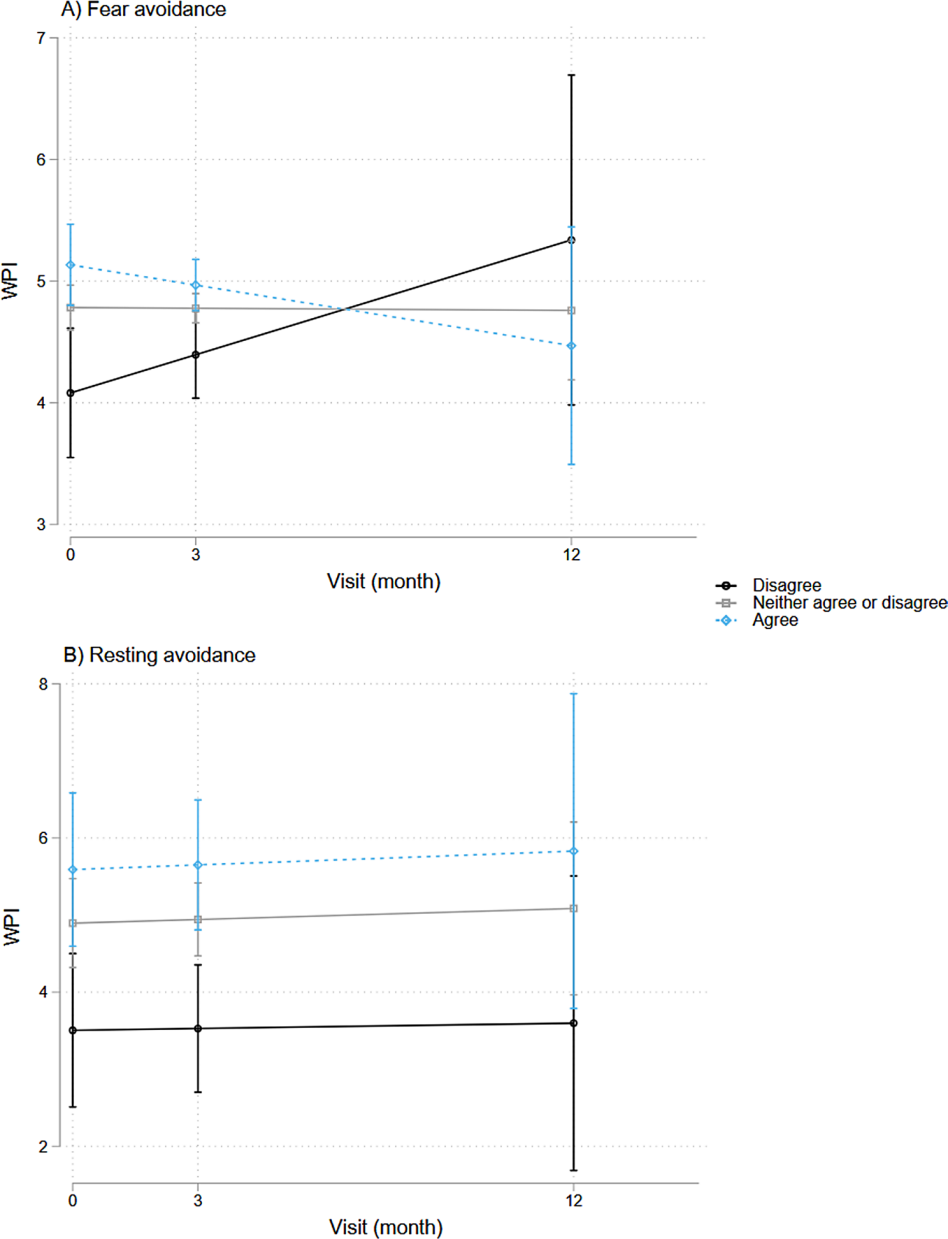
Associations between WPI and baseline coping strategies

Using LGC models, we also examined how baseline coping patterns associated with pain levels at baseline (represented by I) and longitudinally (represented by S) and found several significant associations, depicted in Figs. 6 and 7.

As shown in Fig. 6, higher pain VAS scores associated with certain coping strategies (assessed at baseline), specifically fear avoidance behavior (β-coefficient = 3.0, *p* < 0.001, 95%CI = [1.7, 4.2]), symptom focusing behavior (β-coefficient = 2.2, *p* = 0.002, 95%CI = [0.8, 3.7), embarrassment avoidance behavior (β-coefficient = 2.3, *p* < 0.001, 95%CI = [1.2, 3.4]), and all-or-nothing behavior (β-coefficient = 2.5, *p* < 0.001, 95%CI = [1.1, 3.9]). Higher WPI associated with fear avoidance behavior (β-coefficient = 0.4, *p* < 0.001, 95%CI = [0.2, 0.5]) and embarrassment avoidance behavior (β-coefficient = 0.2, *p* = 0.04, 95%CI = [0.01, 0.3]) (Fig. 7). There were no significant associations with other measured CBRQ subscales in this cohort.

Over time, persistent symptoms were also associated with specific coping strategies (Figs. 6 and 7). Over time, higher pain VAS scores associated significantly with fear avoidance behavior (β-coefficient = 2.1, *p* = 0.015, 95%CI = [0.4, 3.7]), embarrassment avoidance behavior (β-coefficient = 2.5, *p* < 0.001, 95%CI = [1.0, 4.0]), and all-or-nothing behavior (β-coefficient = 2.2, *p* < 0.001, 95%CI = [0.6, 3.9]). On the other hand, higher WPI scores were associated with only embarrassment avoidance behavior over time (β-coefficient = 0.3, *p* = 0.022, 95%CI = [0.04, 0.5]).

### Mediating effect of behavioral responses on the relationship between PHQ9 & GAD7 and pain symptoms

As shown in Table 3, our cross-sectional mediation analyses showed that fear avoidance was a significant mediating factor between PHQ9 and pain VAS scores at baseline, with an indirect effect explaining 28.7% of the total effect. Fear avoidance also mediated GAD7’s effect on pain VAS scores at baseline, with an indirect effect accounting for 42.1% of the total effect. There was no significant mediation effect over time of other coping behaviors at baseline, or when examining PHQ/GAD7’s effect on WPI (Supplementary Tables S3-S12).

**Table 3:** Mediating effects of coping strategies on PHQ9, GAD7 and pain symptoms at baseline

Y	X	M	Estimates	a	b	c’	ab	c	Mediation effect
Pain VAS	PHQ9	Fear avoidance	Coefficient	0.14	2.7	0.97	0.39	1.36	28.7%
			SE	0.04	0.73	0.33	0.15	0.31	
			p value	< 0.001	< 0.001	0.003	0.014	< 0.001	
			95% CI	[0.07, 0.21]	[1.22, 4.17]	[0.32, 1.62]	[0.08, 0.69]	[0.74, 1.97]	
Pain VAS	GAD7	Fear avoidance	Coefficient	0.15	3.01	0.62	0.45	1.07	42.1%
			SE	0.04	0.76	0.39	0.17	0.37	
			p value	< 0.001	< 0.001	0.114	0.011	0.005	
			95% CI	[0.07, 0.23]	[1.52, 4.51]	[-0.12, 1.36]	[0.11, 0.80]	[0.34, 1.80]	

**a = a-path coefficient; b = b-path coefficient; ab = indirect effect; c’ = direct effect; c = total effect

*Y = dependent variable; X = independent variable; M = mediator variable;

## Discussion

This DMARD-switching RA cohort reported moderately high joint pain and widespread pain at baseline. Despite treatment change, these pain symptoms persisted at similar intensity over 12 months, with few patients exhibiting clinically meaningful improvement (using MCID comparable to published thresholds [22, 23]). These trajectories associated with socioeconomic and demographic factors, along with depression and anxiety symptoms, unhelpful appraisals and behavioral responses to somatic symptoms. Importantly, behavioral responses reflecting unhelpful coping strategies mediated the relationship between poor mental health and pain outcomes, suggesting a mechanistic pathway for intervention for affected patients.

The persistent pattern of pain symptoms is consistent with outcomes from clinical trials examining DMARD pain relief efficacy, e.g. approximately 50% of patients starting baricitinib maintained pain VAS scores of > 40 mm(/100 mm) over 6 months [24]. Meta-analyses show that JAK inhibitors improve pain VAS ratings modestly by 4 mm(/100 mm) compared to bDMARDs and by 15 mm compared to placebo, while IL6-inhibiting bDMARDs demonstrated slightly larger effects (-11 mm) than TNF-inhibitors [25, 26]. These similar pain patterns across different therapies suggest a broader limitation beyond individual drug mechanisms. Despite DMARDs effectively suppressing inflammation, they have limited impact on pain mechanisms operating independently of presumed immunological pathways [27].

Similarly to our cohort, limited improvements in disease activity have been reported across DMARD studies and dose escalation schemes [25, 28], though our cohort had fewer patients with good EULAR treatment response in comparison [29]. While this may reflect persistent challenges unique to this cohort, our findings illustrate the insufficient symptom relief patients experience when switching DMARDs. This raises concerns about conventional “treat-to-target” approaches in persistent forms of RA, e.g. difficult-to-treat RA, where physician-assessed inflammatory control often takes priority over patient-centered symptom burden [30]. This discordance can build frustration and undermine treatment engagement [31], stressing the critical need to understand psychological, behavioral, and socioeconomic drivers of persistent symptoms.

### Socioeconomic descriptors and pain outcomes

Pain outcomes were associated with several socioeconomic factors in this cohort, including male gender, financial insecurity, lower education, and employment, representing the social dimension of the biopsychosocial model’s relevance to RA pain management. In this cohort, male gender was associated with higher pain ratings compared to female gender. This was unexpected as there is robust evidence supporting higher pain levels and experience worse RA-related disability in female patients [32]. While we recognize the understudy of the male gender identity in RA [33, 34], it is important to note that our cohort recruited few male patients and there may have been selection bias toward male patients with more severe symptoms, thus warranting cautious interpretation of these results.

Beyond gender, we found significant associations of pain symptoms with socioeconomic status, employment, and education. Social inequality plays a key role in long-term disability and symptom management in RA, so it is unsurprising that patients burdened with financial stress and insecurity reported higher pain ratings. RA is a physically disabling condition that significantly impairs mobility, often forcing patients to work at reduced capacities or leave work [35]. Our cohort showed a positive association between unemployment and widespread pain, consistent with other findings [36], likely reflecting both the disabling effects of widespread pain and symptom exacerbation by additional non-articular pain [5]. Job insecurity, especially with limited economic reserves, can heighten financial stress that reduce the ability to cope with RA symptoms, restricting engagement in helpful activities, thus creating a vicious cycle of increasing strain and worsening symptoms [37].

Additionally, increased pain ratings associated with lower education level, consistent with other studies [38]. This may relate to line of work as employment rates are reduced in patients with lower education, likely due to factors including reduced flexibility, higher physical workload intensity, and reduced disability accommodation among others [39]. Financial strain can further limit the ability to feel “in control” and self-regulate, especially when symptoms recur or worsen [40, 41]. Moreover, the ability and motivation for better self-care are linked to good health literacy, which may rely on the ability to acquire and digest information needed to learn and engage in adaptive coping strategies [42, 43]. While incompletely understood, the mechanisms by which higher education protect against RA symptoms are likely multifactorial. Rather than predictors, lower socioeconomic status and education level should be considered as a proxy for a wide array of factors linked to poor health outcomes. Clinicians should recognize these factors and their potential impact on patients to tailor their support for them. Healthcare services should promote accessible patient resources and provide additional guidance for those who need them.

### Disease activity and pain outcomes

In this RA cohort, baseline disease activity positively associated with higher pain ratings and widespread pain over time, consistent with other reports suggesting baseline DAS28 (especially TJC) predicts severe persistent pain [12]. However, despite this association, DAS28 accounted for minimal variance in changes in pain outcomes, particularly for widespread pain, suggesting that it captures fundamentally different aspects compared to pain VAS and WPI. Importantly, WPI and DAS28 show near-zero overlap in shared variance, highlighting the distinct nature of widespread pain in RA.

This divergence is consistent with other reports of disparities between physician and patient perceptions of symptom severity. Physician assessments are often more influenced by measures of inflammation (e.g. SJC, CRP), whereas patient concerns are more driven by their pain experience and disability [44, 45]. As such, patients may continue to report high pain levels even when inflammatory markers improve, as their pain may no longer be driven by inflammation but rather maintained by factors not captured in conventional disease activity assessments [25]. The decoupled relationship between DAS28 and pain outcomes emphasizes the need to consider additional contributors to persistent pain in RA, including psychosocial and behavioral determinants of health. This also questions the continued reliance on DAS28 scores solely when assessing symptom burden in RA patients. As most patients in this cohort had established disease, the evidence points to an evolving nature of pain origins over time, highlighting the need for multifaceted assessments that captures inflammatory and non-inflammatory components to optimize symptom management.

### Psychological distress and cognitive and behavioral responses

There is compelling evidence that psychological distress is a robust predictor for persistent pain and widespread pain. In this cohort, depression and anxiety symptoms associated positively with joint pain and widespread pain at baseline and longitudinally. These findings are consistent with extensive literature showing comorbid depression and anxiety in chronic pain conditions, including RA, associate with greater pain severity, more unexplained pain, and worse health outcomes [46]. Depression and anxiety are highly prevalent among RA patients and contribute to a cascade of negative effects, including worse prognosis, reduced treatment response, greater disability, and lower quality of life and work [47–49].

Several mechanisms link pain and psychological states across domains of anatomical, cognitive, emotional, and behavioral pathways. As a musculoskeletal condition, the chronic physical symptoms of RA can alter peripheral and central neural processing [50]. In our cohort, the disconnect between DAS28 and pain levels, which instead associated strongly with depression and anxiety symptoms, suggests that pain is increasingly driven by cortical areas that govern pain perception while simultaneously regulating motivational, emotional, and cognitive factors. Extensive psychophysical and neuroimaging studies highlight that the shared neural circuitry of these central mechanisms is increasingly implicated in the relationship between persistent pain and psychological distress [51–55]. This may explain why high levels of depression and anxiety often co-occur with pain persistence and are unlikely to be adequately managed by DMARDs alone [11, 56].

Beyond the shared neurobiological foundation, psychological distress also influences pain via behavioral and cognitive processes, as evidenced by the positive associations between depression/anxiety symptoms and unhelpful cognitive and behavioral patterns. Adaptation to pain is partially governed by the reward circuitry, which shape motivation, symptom perception, and coping behaviors [57]. Patients with higher levels of depression and anxiety may be more likely to catastrophize symptoms or feel a diminished sense of control, which can lead to avoidance of beneficial physical and social activities and poor adherence to treatment recommendations [47]. In this RA cohort, several avoidance-based coping behaviors associated with higher pain ratings and widespread pain, and, particularly, fear avoidance mediated the relationship between depression/anxiety symptoms and pain outcomes. This suggests a key behavioral mechanism maintaining the association between psychological distress and pain symptoms. Such avoidance behavior often carries anxiety and excessive symptom preoccupation, creating positive feedback loops that reinforce safety behaviors and other unhelpful coping patterns that maintain a vicious cycle of fear, passiveness, and worsening pain symptoms [14]. Negative affect and disrupted reward processing may undermine engagement in adaptive coping strategies including physical activity, social interaction, and healthy lifestyles. In the context of RA where patients may experience limited relief from treatment, the compound effect of poor physical and mental health symptoms can further entrench patterns of resting, withdrawal, and isolation. Over time, this can reduce social and familial support, amplify loneliness, and lead to further physical and psychological decline [41].

Cognitive appraisal of symptoms and engagement in active coping patterns are crucial determinants of improved long-term pain outcomes in RA patients. Clinicians should recognize the urgency of providing timely mental health support and accessible patient education, especially at critical clinical decision time points of changing medication, to reduce further risk of engaging in vicious cycles of pain and distress.

### Limitations

This study has several important limitations. First, there were substantial amounts of missing data, particularly at follow-up visits, which may have introduced bias and reduced the precision of our estimates. Thus, the study was also underpowered to detect smaller effect sizes, especially longitudinally, and therefore we were limited in our confidence to draw definitive conclusions about some associations. Secondly, our distribution-derived MCIDs for WPI and pain VAS may not generalize to broader RA populations and are not intended to be applied outside of the study sample. Additionally, information about occupations or line of work was not captured, which may confound some associations as physical strain can impact both pain outcomes and treatment adherence. Furthermore, the recruitment strategy may not have yielded a representative sample, potentially limiting the generalizability of our findings to the broader population of RA patients undergoing treatment change. Finally, causal inference was not possible in this observational study design.

## Conclusion

In conclusion, these findings highlight the importance of applying the biopsychosocial framework in RA management. Biological factors (e.g. disease activity), psychological factors (depression, anxiety, appraisal, coping), and socioeconomic factors (gender, employment, financial strain) collectively influence the persistent pain experience. This matrix of factors reflects a syndemic pattern, where interacting conditions amplify symptom burden beyond their individual effects. Effective care requires an integrative approach that addresses psychological and behavioral factors to support mental health and symptom management strategies, alongside treating inflammation-driven symptoms.

## Supplementary Information

Below is the link to the electronic supplementary material.

Supplementary Material 1

## Data Availability

Data used in this study were collected for PROsPer-RA and are available from the corresponding author on reasonable request. All data relevant to the study are included in the article and its supplementary materials. All figures and tables are original.

## Abbreviations

RA: Rheumatoid arthritis
PROsPer-RA: Patient-Reported Outcomes in patients with Persistent Rheumatoid Arthritis
DMARD: Disease-modifying anti-rheumatic drugs
DAS28: Disease Activity Score in 28 joints
TJC: Tender joint count
SJC: Swollen joint count
CRP: C-reactive protein
VAS: Visual analog scale
PtGA: Patient global assessment
WPI: Widespread pain index
PHQ: Patient Health Questionnaire
GAD: Generalized Anxiety Disorder questionnaire
CBRQ: Cognitive and Behavioral Response Questionnaire
IL: Interleukin
JAK: Janus kinase
