# Supplementary material for: Psychosocial and behavioral correlates of persistent pain post disease-modifying treatment change in rheumatoid arthritis: a 12-month cohort study
Source: Adv Rheumatol. 2026 Mar 31;66:33. doi: 10.1186/s42358-026-00540-4 (PMC13167811; doi:10.1186/s42358-026-00540-4)
Supplement: Supplementary file 1 [file 42358_2026_540_MOESM1_ESM.docx]

# Supplementary Materials

| **Supplementary Table S1: LGC results for pain VAS as outcome.** | | | | | | | | | |
| --- | --- | --- | --- | --- | --- | --- | --- | --- | --- |
| **Predictor** | **Baseline I** | | | **S** | | | **Persistent I** | | |
|  | **Coeff** | **p-value** | **95% CI** | **Coeff** | **p-value** | **95% CI** | **Coeff** | **p-value** | **95% CI** |
| Age | 0.04 | 0.743 | [-0.22, 0.30] | 0.03 | 0.129 | [-0.01, 0.07] | 0.24 | 0.108 | [-0.05, 0.52] |
| Female | -9.46 | 0.034 | [-18.22, -0.70] | 1.14 | 0.077 | [-0.12, 2.4] | -8.86 | 0.066 | [-18.30, 0.57] |
| White | -2.95 | 0.591 | [-13.70, 7.81] | 0.31 | 0.689 | [-1.21, 1.83] | 6.13 | 0.331 | [-6.23, 18.49] |
| Financial stress | 5.15 | 0.026 | [0.61, 9.68] | -0.06 | 0.875 | [-0.78, 0.66] | 2.09 | 0.470 | [-3.57, 7.74] |
| In work | -2.68 | 0.513 | [-10.70, 5.35] | 0.14 | 0.859 | [-1.43, 1.72] | -2.91 | 0.549 | [-12.42, 6.61] |
| Higher education | -10.80 | 0.006 | [-18.54, -3.06] | 0.43 | 0.467 | [-0.73, 1.59] | -4.72 | 0.301 | [-13.66, 4.22] |
| RDCI | 0.26 | 0.886 | [-3.24, 3.76] | 0.40 | 0.134 | [-0.12, 0.92] | 2.89 | 0.124 | [-0.79, 6.57] |
| DAS28 | 7.96 | <0.001 | [5.73, 10.19] | -0.09 | 0.712 | [-0.56, 0.38] | 7.19 | <0.001 | [4.44, 9.93] |
| TJC | 9.59 | <0.001 | [7.19, 11.98] | 0.07 | 0.782 | [-0.44, 0.58] | 9.71 | <0.001 | [6.78, 12.63] |
| SJC | 4.83 | <0.001 | [1.67, 7.98] | -0.23 | 0.562 | [-1.01, 0.55] | 4.07 | 0.002 | [1.48, 6.66] |
| CRP | 1.88 | 0.006 | [0.54, 3.22] | 0.02 | 0.858 | [-0.17, 0.20] | 1.56 | 0.033 | [0.13, 2.99] |
| Seropositivity | 2.68 | 0.415 | [-3.81, 9.17] | -0.18 | 0.751 | [-1.27, 0.92] | 1.96 | 0.576 | [-4.82, 8.74] |
| MH treatment | -1.31 | 0.729 | [-8.84, 6.22] | -0.18 | 0.779 | [-1.45, 1.09] | -2.25 | 0.574 | [-10.30, 5.80] |
| Steroid use | 1.43 | 0.688 | [-5.59, 8.45] | 0.41 | 0.508 | [-0.81, 1.63] | 2.22 | 0.547 | [-5.16, 9.60] |
| Opioid use | 4.09 | 0.257 | [-3.01, 11.19] | 0.39 | 0.541 | [-0.87, 1.65] | 3.53 | 0.365 | [-4.19, 11.25] |
| PHQ9 | 1.52 | <0.001 | [1.09, 1.95] | -0.02 | 0.508 | [-0.08, 0.04] | 1.45 | <0.001 | [0.94, 1.95] |
| GAD7 | 1.24 | <0.001 | [0.74, 1.74] | -0.02 | 0.516 | [-0.09, 0.04] | 1.11 | <0.001 | [0.54, 1.67] |
| Fear avoidance | 0.44 | <0.001 | [0.30, 0.58] | -0.01 | 0.563 | [-0.03, 0.02] | 0.43 | <0.001 | [0.26, 0.59] |
| Damage belief | 0.72 | <0.001 | [0.46, 0.98] | -0.01 | 0.659 | [-0.05, 0.03] | 0.66 | <0.001 | [0.34, 0.97] |
| Symptom focusing | -0.55 | <0.001 | [-0.81, -0.28] | 0.01 | 0.665 | [-0.03, 0.05] | -0.51 | <0.001 | [-0.83, -0.19] |
| Embarrassment avoidance | -0.18 | 0.239 | [-0.48, 0.12] | 0.01 | 0.667 | [-0.03, 0.05] | -0.19 | 0.238 | [-0.51, 0.13] |
| All-or-nothing | 0.81 | <0.001 | [0.49, 1.13] | -0.01 | 0.653 | [-0.05, 0.03] | 0.77 | <0.001 | [0.40, 1.14] |
| Resting avoidance | -0.30 | 0.074 | [-0.63, 0.03] | 0.02 | 0.403 | [-0.02, 0.06] | -0.31 | 0.086 | [-0.67, 0.05] |
| *Baseline I = intercept; S = slope (rate of change over time); Persistent I = persistent shared level across follow-up time points | | | | | | | | | |
| **MH treatment = history of mental health treatment; TJC and SJC were square-root transformed and CRP was log-transformed | | | | | | | | | |

| **Supplementary Table S2: LGC results for WPI as outcome.** | | | | | | | | | |
| --- | --- | --- | --- | --- | --- | --- | --- | --- | --- |
| **Predictor** | **Baseline I** | | | **S** | | | **Persistent I** | | |
|  | **Coeff** | **p-value** | **95% CI** | **Coeff** | **p-value** | **95% CI** | **Coeff** | **p-value** | **95% CI** |
| Age | 0.01 | 0.549 | [-0.02, 0.04] | 0.00 | 0.883 | [-0.004, 0.005] | 0.02 | 0.353 | [-0.02, 0.06] |
| Female | -0.69 | 0.288 | [-1.96, 0.58] | 0.09 | 0.253 | [-0.06, 0.24] | -0.93 | 0.174 | [-2.29, 0.42] |
| White | -0.39 | 0.605 | [-1.80, 1.05] | 0.04 | 0.661 | [-0.15, 0.24] | 0.05 | 0.955 | [-1.55, 1.65] |
| Financial insecure | 0.35 | 0.379 | [-0.43, 1.13] | -0.01 | 0.878 | [-0.11, 0.09] | 0.12 | 0.787 | [-0.75, 0.99] |
| In work | -0.27 | 0.593 | [-1.22, 0.70] | 0.01 | 0.861 | [-0.13, 0.15] | -0.23 | 0.686 | [-1.28, 0.84] |
| Higher education | -0.84 | 0.090 | [-1.82, 0.13] | 0.03 | 0.673 | [-0.10, 0.16] | -0.37 | 0.473 | [-1.40, 0.65] |
| RDCI | 0.15 | 0.554 | [-0.35, 0.65] | 0.03 | 0.301 | [-0.03, 0.09] | 0.35 | 0.175 | [-0.16, 0.86] |
| DAS28 | 0.86 | <0.001 | [0.60, 1.12] | -0.01 | 0.677 | [-0.06, 0.04] | 0.82 | <0.001 | [0.52, 1.12] |
| TJC | 0.93 | <0.001 | [0.61, 1.26] | 0.02 | 0.574 | [-0.05, 0.08] | 0.98 | <0.001 | [0.60, 1.36] |
| SJC | 0.46 | <0.001 | [0.12, 0.80] | -0.02 | 0.605 | [-0.10, 0.06] | 0.42 | 0.015 | [0.08, 0.76] |
| CRP | 0.18 | 0.010 | [0.04, 0.32] | 0.001 | 0.914 | [-0.018, 0.020] | 0.15 | 0.053 | [-0.002, 0.31] |
| Seropositivity | 0.31 | 0.352 | [-0.35, 0.97] | -0.02 | 0.747 | [-0.13, 0.09] | 0.24 | 0.485 | [-0.44, 0.93] |
| MH treatment | -0.13 | 0.730 | [-0.85, 0.59] | -0.02 | 0.779 | [-0.14, 0.11] | -0.20 | 0.606 | [-0.97, 0.57] |
| Steroids | 0.11 | 0.776 | [-0.66, 0.88] | 0.04 | 0.544 | [-0.09, 0.17] | 0.20 | 0.598 | [-0.53, 0.92] |
| Opioids | 0.35 | 0.399 | [-0.46, 1.16] | 0.04 | 0.559 | [-0.10, 0.18] | 0.30 | 0.473 | [-0.52, 1.12] |
| PHQ9 | 0.16 | <0.001 | [0.10, 0.21] | -0.002 | 0.547 | [-0.008, 0.004] | 0.15 | <0.001 | [0.08, 0.21] |
| GAD7 | 0.13 | <0.001 | [0.07, 0.19] | -0.002 | 0.539 | [-0.009, 0.005] | 0.11 | <0.001 | [0.05, 0.18] |
| Fear avoidance | 0.05 | <0.001 | [0.03, 0.07] | -0.001 | 0.576 | [-0.003, 0.002] | 0.05 | <0.001 | [0.02, 0.07] |
| Damage belief | 0.08 | <0.001 | [0.05, 0.11] | -0.001 | 0.684 | [-0.005, 0.003] | 0.07 | <0.001 | [0.03, 0.11] |
| Symptom focusing | -0.06 | <0.001 | [-0.09, -0.03] | 0.001 | 0.686 | [-0.003, 0.005] | -0.05 | 0.001 | [-0.09, -0.02] |
| Embarrassment avoidance | -0.02 | 0.235 | [-0.05, 0.01] | 0.001 | 0.686 | [-0.003, 0.005] | -0.02 | 0.254 | [-0.06, 0.02] |
| All-or-nothing | 0.09 | <0.001 | [0.05, 0.12] | -0.001 | 0.681 | [-0.005, 0.003] | 0.08 | <0.001 | [0.04, 0.12] |
| Resting avoidance | -0.03 | 0.084 | [-0.07, 0.004] | 0.002 | 0.423 | [-0.002, 0.006] | -0.03 | 0.113 | [-0.07, 0.008] |
| *Baseline I = intercept; S = slope (rate of change over time); Persistent I = persistent shared level across follow-up time points | | | | | | | | | |
| **MH treatment = history of mental health treatment; TJC and SJC were square-root transformed and CRP was log-transformed | | | | | | | | | |

| **Supplementary Table S3: Mediating effect of CBRQ variables on PHQ9, GAD7 and pain VAS at baseline.** | | | | | | | | | |
| --- | --- | --- | --- | --- | --- | --- | --- | --- | --- |
| **Y** | **X** | **M** | **Estimates** | **a** | **b** | **c'** | **ab** | **c** | **Mediation** |
| Pain VAS | PHQ9 | Fear avoidance | Coefficient | 0.14 | 2.70 | 0.97 | 0.39 | 1.36 | 28.0% |
|  |  |  | SE | 0.04 | 0.73 | 0.33 | 0.15 | 0.31 |  |
|  |  |  | p value | <0.001 | <0.001 | 0.003 | 0.011 | <0.001 |  |
|  |  |  | 95% CI | [0.07, 0.21] | [1.28, 4.12] | [0.32, 1.62] | [0.09, 0.68] | [0.76, 1.95] |  |
|  |  | Damage belief | Coefficient | 0.06 | 0.54 | 1.32 | 0.03 | 1.35 | 2.0% |
|  |  |  | SE | 0.03 | 1.13 | 0.31 | 0.06 | 0.31 |  |
|  |  |  | p value | 0.094 | 0.633 | <0.001 | 0.618 | <0.001 |  |
|  |  |  | 95% CI | [-0.01, 0.13] | [-1.67, 2.75] | [0.71, 1.93] | [-0.09, 0.16] | [0.75, 1.96] |  |
|  |  | Symptom focusing | Coefficient | 0.10 | 1.69 | 1.20 | 0.17 | 1.37 | 13.0% |
|  |  |  | SE | 0.04 | 0.89 | 0.32 | 0.10 | 0.31 |  |
|  |  |  | p value | 0.009 | 0.058 | <0.001 | 0.097 | <0.001 |  |
|  |  |  | 95% CI | [0.03, 0.18] | [-0.05, 3.43] | [0.57, 1.83] | [-0.03, 0.38] | [0.76, 1.98] |  |
|  |  | Embarrassment avoidance | Coefficient | 0.16 | 1.76 | 1.09 | 0.27 | 1.36 | 20.0% |
|  |  |  | SE | 0.05 | 0.81 | 0.34 | 0.14 | 0.30 |  |
|  |  |  | p value | 0.001 | 0.031 | 0.002 | 0.056 | <0.001 |  |
|  |  |  | 95% CI | [0.07, 0.24] | [0.16, 3.35] | [0.42, 1.77] | [-0.01, 0.55] | [0.77, 1.96] |  |
|  |  | All-or-nothing | Coefficient | 0.13 | 1.54 | 1.17 | 0.20 | 1.37 | 15.0% |
|  |  |  | SE | 0.05 | 0.86 | 0.33 | 0.13 | 0.31 |  |
|  |  |  | p value | 0.008 | 0.074 | <0.001 | 0.102 | <0.001 |  |
|  |  |  | 95% CI | [0.03, 0.22] | [-0.14, 3.22] | [0.53, 1.81] | [-0.04, 0.44] | [0.76, 1.98] |  |
|  |  | Resting avoidance | Coefficient | 0.15 | 0.03 | 1.35 | 0.00 | 1.36 | 0.3% |
|  |  |  | SE | 0.05 | 1.15 | 0.31 | 0.18 | 0.31 |  |
|  |  |  | p value | 0.001 | 0.979 | <0.001 | 0.979 | <0.001 |  |
|  |  |  | 95% CI | [0.06, 0.24] | [-2.22, 2.28] | [0.74, 1.96] | [-0.35, 0.36] | [0.75, 1.96] |  |
| Pain VAS | GAD7 | Fear avoidance | Coefficient | 0.15 | 3.01 | 0.62 | 0.45 | 1.07 | 42.0% |
|  |  |  | SE | 0.04 | 0.76 | 0.39 | 0.17 | 0.37 |  |
|  |  |  | p value | <0.001 | <0.001 | 0.114 | 0.011 | 0.005 |  |
|  |  |  | 95% CI | [0.07, 0.23] | [1.52, 4.51] | [-0.12, 1.36] | [0.11, 0.80] | [0.34, 1.80] |  |
|  |  | Damage belief | Coefficient | 0.10 | 0.47 | 1.03 | 0.05 | 1.08 | 5.0% |
|  |  |  | SE | 0.04 | 1.17 | 0.37 | 0.07 | 0.37 |  |
|  |  |  | p value | 0.009 | 0.690 | 0.005 | 0.441 | 0.004 |  |
|  |  |  | 95% CI | [0.03, 0.18] | [-1.82, 2.76] | [0.31, 1.75] | [-0.08, 0.18] | [0.35, 1.80] |  |
|  |  | Symptom focusing | Coefficient | 0.14 | 1.78 | 0.85 | 0.25 | 1.09 | 23.0% |
|  |  |  | SE | 0.04 | 0.92 | 0.38 | 0.14 | 0.37 |  |
|  |  |  | p value | 0.001 | 0.054 | 0.026 | 0.080 | 0.004 |  |
|  |  |  | 95% CI | [0.06, 0.22] | [-0.02, 3.58] | [0.10, 1.60] | [-0.03, 0.53] | [0.36, 1.81] |  |
|  |  | Embarrassment avoidance | Coefficient | 0.24 | 1.86 | 0.63 | 0.45 | 1.09 | 42.0% |
|  |  |  | SE | 0.06 | 0.85 | 0.40 | 0.17 | 0.36 |  |
|  |  |  | p value | <0.001 | 0.030 | 0.117 | 0.010 | 0.003 |  |
|  |  |  | 95% CI | [0.12, 0.35] | [0.19, 3.53] | [-0.16, 1.42] | [0.11, 0.79] | [0.38, 1.80] |  |
|  |  | All-or-nothing | Coefficient | 0.10 | 2.04 | 0.90 | 0.21 | 1.10 | 19.0% |
|  |  |  | SE | 0.05 | 0.89 | 0.38 | 0.13 | 0.37 |  |
|  |  |  | p value | 0.032 | 0.023 | 0.018 | 0.094 | 0.003 |  |
|  |  |  | 95% CI | [0.01, 0.19] | [0.30, 3.78] | [0.15, 1.65] | [-0.04, 0.45] | [0.37, 1.83] |  |
|  |  | Resting avoidance | Coefficient | 0.13 | 0.53 | 1.02 | 0.07 | 1.08 | 6.0% |
|  |  |  | SE | 0.05 | 1.19 | 0.37 | 0.15 | 0.37 |  |
|  |  |  | p value | 0.007 | 0.656 | 0.006 | 0.630 | 0.004 |  |
|  |  |  | 95% CI | [0.04, 0.23] | [-1.80, 2.86] | [0.29, 1.75] | [-0.23, 0.38] | [0.35, 1.81] |  |

| **Supplementary Table S4: Mediating effect of CBRQ variables on PHQ9, GAD7 and WPI at baseline.** | | | | | | | | | |
| --- | --- | --- | --- | --- | --- | --- | --- | --- | --- |
| **Y** | **X** | **M** | **Estimates** | **a** | **b** | **c'** | **ab** | **c** | **Mediation** |
| WPI | PHQ9 | Fear avoidance | Coefficient | 0.15 | 0.26 | 0.13 | 0.04 | 0.17 | 23.00% |
|  |  |  | SE | 0.04 | 0.07 | 0.03 | 0.02 | 0.03 |  |
|  |  |  | p value | <0.001 | <0.001 | <0.001 | 0.029 | <0.001 |  |
|  |  |  | 95% CI | [0.07, 0.22] | [0.12, 0.40] | [0.07, 0.19] | [0.004, 0.07] | [0.11, 0.23] |  |
|  |  | Damage belief | Coefficient | 0.06 | -0.19 | 0.18 | -0.01 | 0.17 | -7.00% |
|  |  |  | SE | 0.03 | 0.11 | 0.03 | 0.01 | 0.03 |  |
|  |  |  | p value | 0.094 | 0.081 | <0.001 | 0.252 | <0.001 |  |
|  |  |  | 95% CI | [-0.01, 0.13] | [-0.41, 0.02] | [0.12, 0.24] | [-0.03, 0.01] | [0.11, 0.23] |  |
|  |  | Symptom focusing | Coefficient | 0.11 | 0.11 | 0.16 | 0.01 | 0.17 | 7.00% |
|  |  |  | SE | 0.04 | 0.09 | 0.03 | 0.01 | 0.03 |  |
|  |  |  | p value | 0.009 | 0.207 | <0.001 | 0.286 | <0.001 |  |
|  |  |  | 95% CI | [0.03, 0.18] | [-0.06, 0.28] | [0.10, 0.22] | [-0.01, 0.04] | [0.11, 0.23] |  |
|  |  | Embarrassment avoidance | Coefficient | 0.16 | 0.10 | 0.15 | 0.02 | 0.17 | 9.00% |
|  |  |  | SE | 0.05 | 0.08 | 0.03 | 0.01 | 0.03 |  |
|  |  |  | p value | 0.001 | 0.222 | <0.001 | 0.175 | <0.001 |  |
|  |  |  | 95% CI | [0.07, 0.24] | [-0.06, 0.26] | [0.09, 0.21] | [-0.01, 0.04] | [0.11, 0.23] |  |
|  |  | All-or-nothing | Coefficient | 0.14 | 0.02 | 0.17 | 0.00 | 0.17 | 1.00% |
|  |  |  | SE | 0.05 | 0.08 | 0.03 | 0.01 | 0.03 |  |
|  |  |  | p value | 0.008 | 0.802 | <0.001 | 0.805 | <0.001 |  |
|  |  |  | 95% CI | [0.04, 0.23] | [-0.14, 0.18] | [0.11, 0.23] | [-0.02, 0.02] | [0.11, 0.23] |  |
|  |  | Resting avoidance | Coefficient | 0.15 | 0.21 | 0.14 | 0.03 | 0.17 | 18.00% |
|  |  |  | SE | 0.05 | 0.11 | 0.03 | 0.02 | 0.03 |  |
|  |  |  | p value | 0.001 | 0.049 | <0.001 | 0.074 | <0.001 |  |
|  |  |  | 95% CI | [0.06, 0.24] | [0.001, 0.42] | [0.08, 0.20] | [-0.003, 0.07] | [0.11, 0.23] |  |
| WPI | GAD7 | Fear avoidance | Coefficient | 0.16 | 0.28 | 0.12 | 0.05 | 0.16 | 28.00% |
|  |  |  | SE | 0.04 | 0.07 | 0.04 | 0.02 | 0.04 |  |
|  |  |  | p value | <0.001 | <0.001 | 0.001 | 0.006 | <0.001 |  |
|  |  |  | 95% CI | [0.08, 0.24] | [0.14, 0.42] | [0.05, 0.20] | [0.01, 0.08] | [0.09, 0.24] |  |
|  |  | Damage belief | Coefficient | 0.11 | -0.22 | 0.18 | -0.02 | 0.16 | -15.00% |
|  |  |  | SE | 0.04 | 0.12 | 0.04 | 0.02 | 0.04 |  |
|  |  |  | p value | 0.009 | 0.061 | <0.001 | 0.148 | <0.001 |  |
|  |  |  | 95% CI | [0.03, 0.18] | [-0.45, 0.01] | [0.11, 0.26] | [-0.06, 0.01] | [0.09, 0.24] |  |
|  |  | Symptom focusing | Coefficient | 0.15 | 0.12 | 0.14 | 0.02 | 0.16 | 11.00% |
|  |  |  | SE | 0.04 | 0.09 | 0.04 | 0.01 | 0.04 |  |
|  |  |  | p value | 0.001 | 0.194 | <0.001 | 0.203 | <0.001 |  |
|  |  |  | 95% CI | [0.07, 0.23] | [-0.06, 0.30] | [0.07, 0.22] | [-0.01, 0.05] | [0.09, 0.24] |  |
|  |  | Embarrassment avoidance | Coefficient | 0.25 | 0.09 | 0.14 | 0.02 | 0.16 | 14.00% |
|  |  |  | SE | 0.06 | 0.08 | 0.04 | 0.02 | 0.04 |  |
|  |  |  | p value | <0.001 | 0.252 | <0.001 | 0.273 | <0.001 |  |
|  |  |  | 95% CI | [0.13, 0.36] | [-0.07, 0.25] | [0.06, 0.22] | [-0.02, 0.06] | [0.09, 0.24] |  |
|  |  | All-or-nothing | Coefficient | 0.11 | 0.06 | 0.16 | 0.01 | 0.16 | 4.00% |
|  |  |  | SE | 0.05 | 0.09 | 0.04 | 0.01 | 0.04 |  |
|  |  |  | p value | 0.032 | 0.517 | <0.001 | 0.538 | <0.001 |  |
|  |  |  | 95% CI | [0.01, 0.20] | [-0.11, 0.23] | [0.09, 0.24] | [-0.01, 0.03] | [0.09, 0.24] |  |
|  |  | Resting avoidance | Coefficient | 0.13 | 0.25 | 0.13 | 0.03 | 0.16 | 19.00% |
|  |  |  | SE | 0.05 | 1.19 | 0.37 | 0.15 | 0.37 |  |
|  |  |  | p value | 0.007 | 0.656 | 0.006 | 0.630 | 0.004 |  |
|  |  |  | 95% CI | [0.04, 0.23] | [-1.80, 2.86] | [0.29, 1.75] | [-0.23, 0.38] | [0.35, 1.81] |  |

| **Supplementary Table S5: Cross-sectional mediating effect of CBRQ variables on PHQ9, GAD7 and pain VAS (3-mo).** | | | | | | | | | |
| --- | --- | --- | --- | --- | --- | --- | --- | --- | --- |
| **Y** | **X** | **M** | **Estimates** | **a** | **b** | **c'** | **ab** | **c** | **Mediation** |
| Pain VAS | PHQ9 | Fear avoidance | Coefficient | 0.08 | -0.57 | 2.00 | -0.05 | 1.95 | -2.00% |
|  |  |  | SE | 0.07 | 0.99 | 0.53 | 0.09 | 0.55 |  |
|  |  |  | p value | 0.242 | 0.564 | <0.001 | 0.609 | <0.001 |  |
|  |  |  | 95% CI | [-0.05, 0.21] | [-2.50, 1.37] | [0.95, 3.04] | [-0.22, 0.13] | [0.88, 3.03] |  |
|  |  | Damage belief | Coefficient | 0.08 | 3.07 | 1.76 | 0.24 | 1.99 | 12.00% |
|  |  |  | SE | 0.04 | 1.56 | 0.53 | 0.14 | 0.54 |  |
|  |  |  | p value | 0.049 | 0.051 | 0.001 | 0.080 | <0.001 |  |
|  |  |  | 95% CI | [0.001, 0.16] | [-0.01, 6.15] | [0.72, 2.80] | [-0.03, 0.51] | [0.93, 3.05] |  |
|  |  | Symptom focusing | Coefficient | 0.15 | 1.92 | 1.66 | 0.28 | 1.94 | 15.00% |
|  |  |  | SE | 0.05 | 1.21 | 0.54 | 0.16 | 0.55 |  |
|  |  |  | p value | 0.002 | 0.113 | 0.002 | 0.076 | <0.001 |  |
|  |  |  | 95% CI | [0.06, 0.25] | [-0.44, 4.28] | [0.61, 2.72] | [-0.03, 0.59] | [0.87, 3.02] |  |
|  |  | Embarrassment avoidance | Coefficient | 0.22 | 0.34 | 1.88 | 0.07 | 1.95 | 4.00% |
|  |  |  | SE | 0.08 | 1.18 | 0.55 | 0.12 | 0.56 |  |
|  |  |  | p value | 0.005 | 0.774 | 0.001 | 0.548 | <0.001 |  |
|  |  |  | 95% CI | [0.07, 0.37] | [-2.00, 2.67] | [0.79, 2.97] | [-0.16, 0.30] | [0.85, 3.05] |  |
|  |  | All-or-nothing | Coefficient | 0.14 | 0.51 | 1.88 | 0.07 | 1.96 | 4.00% |
|  |  |  | SE | 0.07 | 1.18 | 0.54 | 0.11 | 0.55 |  |
|  |  |  | p value | 0.047 | 0.668 | <0.001 | 0.512 | <0.001 |  |
|  |  |  | 95% CI | [0.002, 0.29] | [-1.81, 2.82] | [0.81, 2.95] | [-0.14, 0.28] | [0.88, 3.03] |  |
|  |  | Resting avoidance | Coefficient | 0.11 | 1.27 | 1.81 | 0.14 | 1.95 | 7.00% |
|  |  |  | SE | 0.06 | 1.63 | 0.54 | 0.15 | 0.55 |  |
|  |  |  | p value | 0.062 | 0.437 | 0.001 | 0.354 | <0.001 |  |
|  |  |  | 95% CI | [-0.005, 0.23] | [-1.92, 4.46] | [0.75, 2.88] | [-0.16, 0.44] | [0.87, 3.03] |  |
| Pain VAS | GAD7 | Fear avoidance | Coefficient | 0.16 | -0.38 | 0.92 | -0.06 | 0.86 | -7.00% |
|  |  |  | SE | 0.07 | 1.04 | 0.67 | 0.10 | 0.69 |  |
|  |  |  | p value | 0.020 | 0.714 | 0.174 | 0.558 | 0.215 |  |
|  |  |  | 95% CI | [0.02, 0.29] | [-2.42, 1.67] | [-0.39, 2.23] | [-0.26, 0.14] | [-0.49, 2.21] |  |
|  |  | Damage belief | Coefficient | 0.08 | 3.70 | 0.62 | 0.29 | 0.92 | 32.00% |
|  |  |  | SE | 0.04 | 1.61 | 0.67 | 0.16 | 0.68 |  |
|  |  |  | p value | 0.080 | 0.023 | 0.355 | 0.070 | 0.178 |  |
|  |  |  | 95% CI | [-0.01, 0.17] | [0.54, 6.86] | [-0.69, 1.93] | [-0.02, 0.60] | [-0.41, 2.25] |  |
|  |  | Symptom focusing | Coefficient | 0.12 | 2.61 | 0.47 | 0.31 | 0.78 | 39.00% |
|  |  |  | SE | 0.05 | 1.25 | 0.68 | 0.17 | 0.69 |  |
|  |  |  | p value | 0.015 | 0.038 | 0.490 | 0.068 | 0.259 |  |
|  |  |  | 95% CI | [0.02, 0.21] | [0.16, 5.06] | [-0.87, 1.81] | [-0.02, 0.64] | [-0.57, 2.13] |  |
|  |  | Embarrassment avoidance | Coefficient | 0.25 | 0.98 | 0.53 | 0.25 | 0.77 | 32.00% |
|  |  |  | SE | 0.08 | 1.21 | 0.69 | 0.15 | 0.70 |  |
|  |  |  | p value | 0.001 | 0.417 | 0.440 | 0.106 | 0.274 |  |
|  |  |  | 95% CI | [0.10, 0.40] | [-1.39, 3.35] | [-0.82, 1.88] | [-0.05, 0.55] | [-0.60, 2.14] |  |
|  |  | All-or-nothing | Coefficient | 0.10 | 2.45 | 0.53 | 0.24 | 0.77 | 31.00% |
|  |  |  | SE | 0.07 | 1.22 | 0.68 | 0.18 | 0.69 |  |
|  |  |  | p value | 0.144 | 0.046 | 0.434 | 0.183 | 0.265 |  |
|  |  |  | 95% CI | [-0.03, 0.23] | [0.06, 4.84] | [-0.81, 1.87] | [-0.12, 0.59] | [-0.58, 2.12] |  |
|  |  | Resting avoidance | Coefficient | 0.11 | 1.82 | 0.58 | 0.20 | 0.78 | 26.00% |
|  |  |  | SE | 0.06 | 1.68 | 0.68 | 0.17 | 0.69 |  |
|  |  |  | p value | 0.057 | 0.280 | 0.394 | 0.242 | 0.257 |  |
|  |  |  | 95% CI | [-0.003, 0.23] | [-1.47, 5.11] | [-0.75, 1.91] | [-0.14, 0.54] | [-0.57, 2.13] |  |

| **Supplementary Table S6: Cross-sectional mediating effect of CBRQ variables on PHQ9, GAD7 and WPI (3-mo).** | | | | | | | | | |
| --- | --- | --- | --- | --- | --- | --- | --- | --- | --- |
| **Y** | **X** | **M** | **Estimates** | **a** | **b** | **c'** | **ab** | **c** | **Mediation** |
| WPI | PHQ9 | Fear avoidance | Coefficient | 0.08 | -0.07 | 0.22 | -0.01 | 0.22 | -3.00% |
|  |  |  | SE | 0.07 | 0.11 | 0.06 | 0.01 | 0.06 |  |
|  |  |  | p value | 0.242 | 0.559 | <0.001 | 0.607 | <0.001 |  |
|  |  |  | 95% CI | [-0.05, 0.21] | [-0.29, 0.16] | [0.10, 0.33] | [-0.03, 0.02] | [0.10, 0.34] |  |
|  |  | Damage belief | Coefficient | 0.08 | 0.39 | 0.19 | 0.03 | 0.22 | 14.00% |
|  |  |  | SE | 0.04 | 0.18 | 0.06 | 0.02 | 0.06 |  |
|  |  |  | p value | 0.049 | 0.033 | 0.002 | 0.073 | <0.001 |  |
|  |  |  | 95% CI | [0.001, 0.16] | [0.03, 0.74] | [0.07, 0.31] | [-0.003, 0.06] | [0.10, 0.34] |  |
|  |  | Symptom focusing | Coefficient | 0.15 | 0.23 | 0.18 | 0.04 | 0.22 | 16.00% |
|  |  |  | SE | 0.05 | 0.14 | 0.06 | 0.02 | 0.06 |  |
|  |  |  | p value | 0.002 | 0.106 | 0.004 | 0.073 | <0.001 |  |
|  |  |  | 95% CI | [0.06, 0.25] | [-0.05, 0.51] | [0.06, 0.30] | [-0.004, 0.08] | [0.10, 0.34] |  |
|  |  | Embarrassment avoidance | Coefficient | 0.22 | 0.04 | 0.21 | 0.01 | 0.22 | 4.00% |
|  |  |  | SE | 0.08 | 0.13 | 0.06 | 0.01 | 0.06 |  |
|  |  |  | p value | 0.005 | 0.772 | 0.001 | 0.548 | <0.001 |  |
|  |  |  | 95% CI | [0.07, 0.37] | [-0.22, 0.30] | [0.09, 0.33] | [-0.02, 0.03] | [0.10, 0.34] |  |
|  |  | All-or-nothing | Coefficient | 0.14 | 0.06 | 0.21 | 0.01 | 0.22 | 4.00% |
|  |  |  | SE | 0.07 | 0.13 | 0.06 | 0.01 | 0.06 |  |
|  |  |  | p value | 0.047 | 0.668 | <0.001 | 0.513 | <0.001 |  |
|  |  |  | 95% CI | [0.002, 0.29] | [-0.20, 0.32] | [0.09, 0.33] | [-0.02, 0.03] | [0.10, 0.34] |  |
|  |  | Resting avoidance | Coefficient | 0.11 | 0.15 | 0.20 | 0.02 | 0.22 | 8.00% |
|  |  |  | SE | 0.06 | 0.18 | 0.06 | 0.02 | 0.06 |  |
|  |  |  | p value | 0.062 | 0.414 | 0.001 | 0.350 | <0.001 |  |
|  |  |  | 95% CI | [-0.005, 0.23] | [-0.21, 0.51] | [0.08, 0.32] | [-0.02, 0.05] | [0.10, 0.34] |  |
| WPI | GAD7 | Fear avoidance | Coefficient | 0.16 | -0.04 | 0.13 | -0.01 | 0.12 | -5.00% |
|  |  |  | SE | 0.07 | 0.11 | 0.07 | 0.01 | 0.07 |  |
|  |  |  | p value | 0.020 | 0.714 | 0.084 | 0.558 | 0.108 |  |
|  |  |  | 95% CI | [0.02, 0.29] | [-0.26, 0.18] | [-0.02, 0.27] | [-0.03, 0.01] | [-0.03, 0.27] |  |
|  |  | Damage belief | Coefficient | 0.08 | 0.47 | 0.09 | 0.04 | 0.13 | 28.00% |
|  |  |  | SE | 0.04 | 0.17 | 0.07 | 0.02 | 0.07 |  |
|  |  |  | p value | 0.080 | 0.007 | 0.210 | 0.064 | 0.081 |  |
|  |  |  | 95% CI | [-0.01, 0.17] | [0.13, 0.81] | [-0.05, 0.23] | [-0.002, 0.08] | [-0.02, 0.28] |  |
|  |  | Symptom focusing | Coefficient | 0.12 | 0.33 | 0.09 | 0.04 | 0.13 | 31.00% |
|  |  |  | SE | 0.05 | 0.13 | 0.07 | 0.02 | 0.07 |  |
|  |  |  | p value | 0.015 | 0.013 | 0.197 | 0.066 | 0.074 |  |
|  |  |  | 95% CI | [0.02, 0.21] | [0.07, 0.59] | [-0.05, 0.23] | [-0.002, 0.08] | [-0.01, 0.27] |  |
|  |  | Embarrassment avoidance | Coefficient | 0.25 | 0.12 | 0.10 | 0.03 | 0.13 | 23.00% |
|  |  |  | SE | 0.08 | 0.13 | 0.07 | 0.02 | 0.07 |  |
|  |  |  | p value | 0.001 | 0.350 | 0.148 | 0.103 | 0.075 |  |
|  |  |  | 95% CI | [0.10, 0.40] | [-0.13, 0.37] | [-0.04, 0.24] | [-0.01, 0.07] | [-0.01, 0.27] |  |
|  |  | All-or-nothing | Coefficient | 0.10 | 0.30 | 0.10 | 0.03 | 0.13 | 23.00% |
|  |  |  | SE | 0.07 | 0.13 | 0.07 | 0.02 | 0.07 |  |
|  |  |  | p value | 0.144 | 0.025 | 0.152 | 0.176 | 0.074 |  |
|  |  |  | 95% CI | [-0.03, 0.23] | [0.04, 0.56] | [-0.04, 0.24] | [-0.01, 0.07] | [-0.01, 0.27] |  |
|  |  | Resting avoidance | Coefficient | 0.11 | 0.22 | 0.11 | 0.02 | 0.14 | 18.00% |
|  |  |  | SE | 0.06 | 0.18 | 0.07 | 0.02 | 0.07 |  |
|  |  |  | p value | 0.057 | 0.227 | 0.120 | 0.237 | 0.058 |  |
|  |  |  | 95% CI | [-0.003, 0.23] | [-0.13, 0.57] | [-0.03, 0.25] | [-0.02, 0.06] | [-0.005, 0.28] |  |

| **Supplementary Table S7: Cross-sectional mediating effect of CBRQ variables on PHQ9, GAD7 and pain VAS (12-mo).** | | | | | | | | | |
| --- | --- | --- | --- | --- | --- | --- | --- | --- | --- |
| **Y** | **X** | **M** | **Estimates** | **a** | **b** | **c'** | **ab** | **c** | **Mediation** |
| Pain VAS | PHQ9 | Fear avoidance | Coefficient | 0.12 | 0.91 | 2.74 | 0.11 | 2.85 | 4.00% |
|  |  |  | SE | 0.04 | 1.28 | 0.59 | 0.15 | 0.56 |  |
|  |  |  | p value | 0.005 | 0.476 | <0.001 | 0.472 | <0.001 |  |
|  |  |  | 95% CI | [0.04, 0.20] | [-1.59, 3.42] | [1.58, 3.91] | [-0.19, 0.40] | [1.75, 3.96] |  |
|  |  | Damage belief | Coefficient | 0.10 | -1.39 | 3.02 | -0.13 | 2.89 | -5.00% |
|  |  |  | SE | 0.04 | 1.63 | 0.57 | 0.17 | 0.56 |  |
|  |  |  | p value | 0.010 | 0.394 | <0.001 | 0.431 | <0.001 |  |
|  |  |  | 95% CI | [0.02, 0.17] | [-4.59, 1.81] | [1.91, 4.14] | [-0.47, 0.20] | [1.78, 4.00] |  |
|  |  | Symptom focusing | Coefficient | 0.14 | 1.13 | 2.74 | 0.15 | 2.89 | 5.00% |
|  |  |  | SE | 0.04 | 1.42 | 0.58 | 0.20 | 0.57 |  |
|  |  |  | p value | 0.001 | 0.427 | <0.001 | 0.453 | <0.001 |  |
|  |  |  | 95% CI | [0.05, 0.22] | [-1.65, 3.91] | [1.60, 3.88] | [-0.24, 0.54] | [1.77, 4.01] |  |
|  |  | Embarrassment avoidance | Coefficient | 0.23 | 1.85 | 2.46 | 0.42 | 2.88 | 15.00% |
|  |  |  | SE | 0.06 | 1.17 | 0.58 | 0.17 | 0.56 |  |
|  |  |  | p value | <0.001 | 0.116 | <0.001 | 0.013 | <0.001 |  |
|  |  |  | 95% CI | [0.11, 0.35] | [-0.44, 4.14] | [1.32, 3.60] | [0.09, 0.75] | [1.78, 3.98] |  |
|  |  | All-or-nothing | Coefficient | 0.12 | 1.89 | 2.67 | 0.23 | 2.90 | 8.00% |
|  |  |  | SE | 0.05 | 1.32 | 0.58 | 0.18 | 0.57 |  |
|  |  |  | p value | 0.011 | 0.152 | <0.001 | 0.197 | <0.001 |  |
|  |  |  | 95% CI | [0.03, 0.22] | [-0.69, 4.47] | [1.53, 3.81] | [-0.12, 0.58] | [1.78, 4.02] |  |
|  |  | Resting avoidance | Coefficient | 0.16 | 0.73 | 2.74 | 0.12 | 2.85 | 4.00% |
|  |  |  | SE | 0.05 | 1.55 | 0.58 | 0.26 | 0.57 |  |
|  |  |  | p value | 0.001 | 0.637 | <0.001 | 0.650 | <0.001 |  |
|  |  |  | 95% CI | [0.06, 0.26] | [-2.31, 3.77] | [1.59, 3.89] | [-0.39, 0.62] | [1.73, 3.98] |  |
| Pain VAS | GAD7 | Fear avoidance | Coefficient | 0.14 | 1.82 | 3.18 | 0.25 | 3.43 | 7.00% |
|  |  |  | SE | 0.04 | 1.37 | 0.77 | 0.20 | 0.74 |  |
|  |  |  | p value | 0.001 | 0.186 | <0.001 | 0.213 | <0.001 |  |
|  |  |  | 95% CI | [0.06, 0.22] | [-0.86, 4.50] | [1.66, 4.70] | [-0.14, 0.64] | [1.97, 4.89] |  |
|  |  | Damage belief | Coefficient | 0.18 | -2.20 | 3.96 | -0.39 | 3.58 | -11.00% |
|  |  |  | SE | 0.05 | 1.73 | 0.75 | 0.32 | 0.75 |  |
|  |  |  | p value | <0.001 | 0.205 | <0.001 | 0.226 | <0.001 |  |
|  |  |  | 95% CI | [0.09, 0.28] | [-5.59, 1.19] | [2.48, 5.44] | [-1.02, 0.24] | [2.11, 5.05] |  |
|  |  | Symptom focusing | Coefficient | 0.20 | 1.38 | 3.29 | 0.28 | 3.57 | 8.00% |
|  |  |  | SE | 0.05 | 1.52 | 0.76 | 0.32 | 0.75 |  |
|  |  |  | p value | <0.001 | 0.364 | <0.001 | 0.379 | <0.001 |  |
|  |  |  | 95% CI | [0.10, 0.29] | [-1.60, 4.36] | [1.80, 4.78] | [-0.35, 0.91] | [2.10, 5.04] |  |
|  |  | Embarrassment avoidance | Coefficient | 0.25 | 2.46 | 2.93 | 0.62 | 3.55 | 18.00% |
|  |  |  | SE | 0.06 | 1.25 | 0.76 | 0.21 | 0.74 |  |
|  |  |  | p value | <0.001 | 0.050 | <0.001 | 0.003 | <0.001 |  |
|  |  |  | 95% CI | [0.13, 0.37] | [0.01, 4.91] | [1.44, 4.42] | [0.21, 1.03] | [2.09, 5.01] |  |
|  |  | All-or-nothing | Coefficient | 0.12 | 2.89 | 3.13 | 0.35 | 3.48 | 10.00% |
|  |  |  | SE | 0.05 | 1.41 | 0.76 | 0.21 | 0.75 |  |
|  |  |  | p value | 0.013 | 0.041 | <0.001 | 0.095 | <0.001 |  |
|  |  |  | 95% CI | [0.03, 0.22] | [0.13, 5.65] | [1.64, 4.62] | [-0.06, 0.76] | [2.01, 4.95] |  |
|  |  | Resting avoidance | Coefficient | 0.18 | 1.46 | 3.23 | 0.26 | 3.49 | 8.00% |
|  |  |  | SE | 0.05 | 1.65 | 0.76 | 0.31 | 0.75 |  |
|  |  |  | p value | <0.001 | 0.377 | <0.001 | 0.402 | <0.001 |  |
|  |  |  | 95% CI | [0.08, 0.28] | [-1.78, 4.70] | [1.74, 4.72] | [-0.35, 0.87] | [2.02, 4.96] |  |

| **Supplementary Table S8: Cross-sectional mediating effect of CBRQ variables on PHQ9, GAD7 and WPI (12-mo).** | | | | | | | | | |
| --- | --- | --- | --- | --- | --- | --- | --- | --- | --- |
| **Y** | **X** | **M** | **Estimates** | **a** | **b** | **c'** | **ab** | **c** | **Mediation** |
| WPI | PHQ9 | Fear avoidance | Coefficient | 0.12 | 0.10 | 0.30 | 0.01 | 0.31 | 4.00% |
|  |  |  | SE | 0.04 | 0.14 | 0.06 | 0.02 | 0.06 |  |
|  |  |  | p value | 0.005 | 0.476 | <0.001 | 0.472 | <0.001 |  |
|  |  |  | 95% CI | [0.04, 0.20] | [-0.17, 0.37] | [0.17, 0.42] | [-0.02, 0.04] | [0.19, 0.43] |  |
|  |  | Damage belief | Coefficient | 0.10 | -0.15 | 0.33 | -0.01 | 0.31 | -4.00% |
|  |  |  | SE | 0.04 | 0.18 | 0.06 | 0.02 | 0.06 |  |
|  |  |  | p value | 0.010 | 0.394 | <0.001 | 0.431 | <0.001 |  |
|  |  |  | 95% CI | [0.02, 0.17] | [-0.50, 0.20] | [0.21, 0.45] | [-0.05, 0.02] | [0.19, 0.43] |  |
|  |  | Symptom focusing | Coefficient | 0.14 | 0.12 | 0.30 | 0.02 | 0.32 | 5.00% |
|  |  |  | SE | 0.04 | 0.15 | 0.06 | 0.02 | 0.06 |  |
|  |  |  | p value | 0.001 | 0.427 | <0.001 | 0.453 | <0.001 |  |
|  |  |  | 95% CI | [0.05, 0.22] | [-0.18, 0.42] | [0.18, 0.42] | [-0.03, 0.06] | [0.20, 0.44] |  |
|  |  | Embarrassment avoidance | Coefficient | 0.23 | 0.20 | 0.27 | 0.05 | 0.31 | 15.00% |
|  |  |  | SE | 0.06 | 0.13 | 0.06 | 0.02 | 0.06 |  |
|  |  |  | p value | <0.001 | 0.116 | <0.001 | 0.013 | <0.001 |  |
|  |  |  | 95% CI | [0.11, 0.35] | [-0.05, 0.45] | [0.15, 0.39] | [0.01, 0.08] | [0.19, 0.43] |  |
|  |  | All-or-nothing | Coefficient | 0.12 | 0.21 | 0.29 | 0.02 | 0.31 | 8.00% |
|  |  |  | SE | 0.05 | 0.14 | 0.06 | 0.02 | 0.06 |  |
|  |  |  | p value | 0.011 | 0.152 | <0.001 | 0.197 | <0.001 |  |
|  |  |  | 95% CI | [0.03, 0.22] | [-0.08, 0.50] | [0.17, 0.42] | [-0.01, 0.06] | [0.19, 0.43] |  |
|  |  | Resting avoidance | Coefficient | 0.16 | 0.08 | 0.30 | 0.01 | 0.31 | 4.00% |
|  |  |  | SE | 0.05 | 0.17 | 0.06 | 0.03 | 0.06 |  |
|  |  |  | p value | 0.001 | 0.637 | <0.001 | 0.650 | <0.001 |  |
|  |  |  | 95% CI | [0.06, 0.26] | [-0.25, 0.41] | [0.17, 0.42] | [-0.04, 0.07] | [0.19, 0.43] |  |
| WPI | GAD7 | Fear avoidance | Coefficient | 0.14 | 0.20 | 0.37 | 0.03 | 0.40 | 7.00% |
|  |  |  | SE | 0.04 | 0.15 | 0.09 | 0.02 | 0.09 |  |
|  |  |  | p value | 0.001 | 0.186 | <0.001 | 0.213 | <0.001 |  |
|  |  |  | 95% CI | [0.06, 0.22] | [-0.09, 0.49] | [0.19, 0.55] | [-0.02, 0.07] | [0.23, 0.57] |  |
|  |  | Damage belief | Coefficient | 0.18 | -0.24 | 0.44 | -0.04 | 0.40 | -11.00% |
|  |  |  | SE | 0.05 | 0.19 | 0.09 | 0.04 | 0.09 |  |
|  |  |  | p value | <0.001 | 0.205 | <0.001 | 0.226 | <0.001 |  |
|  |  |  | 95% CI | [0.09, 0.28] | [-0.61, 0.13] | [0.27, 0.61] | [-0.11, 0.03] | [0.23, 0.57] |  |
|  |  | Symptom focusing | Coefficient | 0.20 | 0.15 | 0.37 | 0.03 | 0.40 | 8.00% |
|  |  |  | SE | 0.05 | 0.17 | 0.09 | 0.04 | 0.09 |  |
|  |  |  | p value | <0.001 | 0.364 | <0.001 | 0.379 | <0.001 |  |
|  |  |  | 95% CI | [0.10, 0.29] | [-0.18, 0.48] | [0.20, 0.54] | [-0.04, 0.10] | [0.23, 0.57] |  |
|  |  | Embarrassment avoidance | Coefficient | 0.25 | 0.27 | 0.33 | 0.07 | 0.40 | 18.00% |
|  |  |  | SE | 0.06 | 0.14 | 0.09 | 0.02 | 0.09 |  |
|  |  |  | p value | <0.001 | 0.050 | <0.001 | 0.003 | <0.001 |  |
|  |  |  | 95% CI | [0.13, 0.37] | [0.001, 0.54] | [0.16, 0.50] | [0.02, 0.11] | [0.23, 0.57] |  |
|  |  | All-or-nothing | Coefficient | 0.12 | 0.32 | 0.36 | 0.04 | 0.40 | 10.00% |
|  |  |  | SE | 0.05 | 0.15 | 0.09 | 0.02 | 0.09 |  |
|  |  |  | p value | 0.013 | 0.041 | <0.001 | 0.095 | <0.001 |  |
|  |  |  | 95% CI | [0.03, 0.22] | [0.01, 0.63] | [0.19, 0.53] | [-0.01, 0.08] | [0.23, 0.57] |  |
|  |  | Resting avoidance | Coefficient | 0.18 | 0.16 | 0.37 | 0.03 | 0.40 | 8.00% |
|  |  |  | SE | 0.05 | 0.18 | 0.09 | 0.03 | 0.09 |  |
|  |  |  | p value | <0.001 | 0.377 | <0.001 | 0.402 | <0.001 |  |
|  |  |  | 95% CI | [0.08, 0.28] | [-0.19, 0.51] | [0.20, 0.54] | [-0.04, 0.10] | [0.23, 0.57] |  |

| **Supplementary Table S9: Longitudinal mediating effect of CBRQ variables on PHQ9, GAD7 and pain VAS (3-mo).** | | | | | | | | | |
| --- | --- | --- | --- | --- | --- | --- | --- | --- | --- |
| **Y** | **X** | **M** | **Estimates** | **a** | **b** | **c'** | **ab** | **c** | **Mediation** |
| Pain VAS | PHQ9 | Fear avoidance | Coefficient | 0.12 | -0.78 | 1.38 | -0.10 | 1.29 | -8.00% |
|  |  |  | SE | 0.05 | 0.81 | 0.34 | 0.11 | 0.35 |  |
|  |  |  | p value | 0.007 | 0.335 | <0.001 | 0.367 | <0.001 |  |
|  |  |  | 95% CI | [0.03, 0.21] | [-2.37, 0.81] | [0.72, 2.05] | [-0.31, 0.11] | [0.60, 1.97] |  |
|  |  | Damage belief | Coefficient | 0.07 | 1.94 | 1.17 | 0.14 | 1.31 | 10.00% |
|  |  |  | SE | 0.03 | 1.20 | 0.34 | 0.09 | 0.35 |  |
|  |  |  | p value | 0.028 | 0.106 | 0.001 | 0.109 | <0.001 |  |
|  |  |  | 95% CI | [0.01, 0.13] | [-0.41, 4.29] | [0.50, 1.84] | [-0.03, 0.31] | [0.63, 1.99] |  |
|  |  | Symptom focusing | Coefficient | 0.17 | 1.36 | 1.05 | 0.24 | 1.28 | 18.00% |
|  |  |  | SE | 0.05 | 0.95 | 0.35 | 0.11 | 0.36 |  |
|  |  |  | p value | 0.001 | 0.154 | 0.003 | 0.028 | <0.001 |  |
|  |  |  | 95% CI | [0.07, 0.26] | [-0.50, 3.22] | [0.36, 1.74] | [0.03, 0.45] | [0.57, 1.99] |  |
|  |  | Embarrassment avoidance | Coefficient | 0.24 | 0.24 | 1.23 | 0.06 | 1.29 | 5.00% |
|  |  |  | SE | 0.06 | 0.89 | 0.35 | 0.08 | 0.36 |  |
|  |  |  | p value | <0.001 | 0.789 | <0.001 | 0.467 | <0.001 |  |
|  |  |  | 95% CI | [0.12, 0.36] | [-1.51, 1.99] | [0.54, 1.92] | [-0.10, 0.22] | [0.58, 2.00] |  |
|  |  | All-or-nothing | Coefficient | 0.12 | 0.44 | 1.24 | 0.06 | 1.29 | 4.00% |
|  |  |  | SE | 0.05 | 0.95 | 0.35 | 0.08 | 0.36 |  |
|  |  |  | p value | 0.015 | 0.642 | <0.001 | 0.489 | <0.001 |  |
|  |  |  | 95% CI | [0.02, 0.23] | [-1.42, 2.30] | [0.55, 1.93] | [-0.11, 0.22] | [0.58, 2.00] |  |
|  |  | Resting avoidance | Coefficient | 0.15 | 1.61 | 1.05 | 0.24 | 1.29 | 19.00% |
|  |  |  | SE | 0.05 | 1.30 | 0.35 | 0.15 | 0.36 |  |
|  |  |  | p value | 0.004 | 0.219 | 0.003 | 0.115 | <0.001 |  |
|  |  |  | 95% CI | [0.05, 0.25] | [-0.94, 4.16] | [0.36, 1.74] | [-0.06, 0.54] | [0.58, 2.00] |  |
| Pain VAS | GAD7 | Fear avoidance | Coefficient | 0.19 | -0.69 | 1.00 | -0.13 | 0.88 | -15.00% |
|  |  |  | SE | 0.05 | 0.82 | 0.41 | 0.11 | 0.42 |  |
|  |  |  | p value | <0.001 | 0.402 | 0.015 | 0.254 | 0.036 |  |
|  |  |  | 95% CI | [0.09, 0.29] | [-2.30, 0.92] | [0.19, 1.81] | [-0.35, 0.09] | [0.06, 1.70] |  |
|  |  | Damage belief | Coefficient | 0.07 | 2.22 | 0.76 | 0.17 | 0.92 | 18.00% |
|  |  |  | SE | 0.04 | 1.24 | 0.41 | 0.11 | 0.42 |  |
|  |  |  | p value | 0.058 | 0.075 | 0.065 | 0.120 | 0.029 |  |
|  |  |  | 95% CI | [-0.002, 0.15] | [-0.21, 4.65] | [-0.04, 1.56] | [-0.04, 0.38] | [0.09, 1.75] |  |
|  |  | Symptom focusing | Coefficient | 0.17 | 1.79 | 0.53 | 0.30 | 0.83 | 36.00% |
|  |  |  | SE | 0.05 | 0.99 | 0.42 | 0.12 | 0.43 |  |
|  |  |  | p value | 0.001 | 0.071 | 0.209 | 0.012 | 0.054 |  |
|  |  |  | 95% CI | [0.07, 0.26] | [-0.15, 3.73] | [-0.29, 1.35] | [0.07, 0.53] | [-0.01, 1.67] |  |
|  |  | Embarrassment avoidance | Coefficient | 0.28 | 0.62 | 0.65 | 0.18 | 0.83 | 21.00% |
|  |  |  | SE | 0.07 | 0.92 | 0.42 | 0.10 | 0.43 |  |
|  |  |  | p value | <0.001 | 0.502 | 0.125 | 0.080 | 0.054 |  |
|  |  |  | 95% CI | [0.14, 0.42] | [-1.18, 2.42] | [-0.18, 1.48] | [-0.02, 0.38] | [-0.01, 1.67] |  |
|  |  | All-or-nothing | Coefficient | 0.11 | 1.50 | 0.67 | 0.16 | 0.84 | 19.00% |
|  |  |  | SE | 0.06 | 0.98 | 0.42 | 0.11 | 0.43 |  |
|  |  |  | p value | 0.065 | 0.126 | 0.114 | 0.135 | 0.050 |  |
|  |  |  | 95% CI | [-0.01, 0.23] | [-0.42, 3.42] | [-0.16, 1.50] | [-0.05, 0.37] | [0.001, 1.68] |  |
|  |  | Resting avoidance | Coefficient | 0.12 | 2.34 | 0.56 | 0.27 | 0.83 | 33.00% |
|  |  |  | SE | 0.06 | 1.35 | 0.42 | 0.17 | 0.43 |  |
|  |  |  | p value | 0.030 | 0.084 | 0.185 | 0.107 | 0.055 |  |
|  |  |  | 95% CI | [0.01, 0.23] | [-0.31, 4.99] | [-0.27, 1.39] | [-0.06, 0.60] | [-0.01, 1.67] |  |

| **Supplementary Table S10: Longitudinal mediating effect of CBRQ variables on PHQ9, GAD7 and WPI (3-mo).** | | | | | | | | | |
| --- | --- | --- | --- | --- | --- | --- | --- | --- | --- |
| **Y** | **X** | **M** | **Estimates** | **a** | **b** | **c'** | **ab** | **c** | **Mediation** |
| WPI | PHQ9 | Fear avoidance | Coefficient | 0.12 | -0.78 | 1.38 | -0.10 | 1.29 | -8.00% |
|  |  |  | SE | 0.05 | 0.81 | 0.34 | 0.11 | 0.35 |  |
|  |  |  | p value | 0.007 | 0.335 | <0.001 | 0.367 | <0.001 |  |
|  |  |  | 95% CI | [0.03, 0.21] | [-2.37, 0.81] | [0.72, 2.05] | [-0.31, 0.11] | [0.60, 1.97] |  |
|  |  | Damage belief | Coefficient | 0.07 | 1.94 | 1.17 | 0.14 | 1.31 | 10.00% |
|  |  |  | SE | 0.03 | 1.20 | 0.34 | 0.09 | 0.35 |  |
|  |  |  | p value | 0.028 | 0.106 | 0.001 | 0.109 | <0.001 |  |
|  |  |  | 95% CI | [0.01, 0.13] | [-0.41, 4.29] | [0.50, 1.84] | [-0.03, 0.31] | [0.63, 1.99] |  |
|  |  | Symptom focusing | Coefficient | 0.17 | 1.36 | 1.05 | 0.24 | 1.28 | 18.00% |
|  |  |  | SE | 0.05 | 0.95 | 0.35 | 0.11 | 0.36 |  |
|  |  |  | p value | 0.001 | 0.154 | 0.003 | 0.028 | <0.001 |  |
|  |  |  | 95% CI | [0.07, 0.26] | [-0.50, 3.22] | [0.36, 1.74] | [0.03, 0.45] | [0.57, 1.99] |  |
|  |  | Embarrassment avoidance | Coefficient | 0.24 | 0.24 | 1.23 | 0.06 | 1.29 | 5.00% |
|  |  |  | SE | 0.06 | 0.89 | 0.35 | 0.08 | 0.36 |  |
|  |  |  | p value | <0.001 | 0.789 | <0.001 | 0.467 | <0.001 |  |
|  |  |  | 95% CI | [0.12, 0.36] | [-1.51, 1.99] | [0.54, 1.92] | [-0.10, 0.22] | [0.58, 2.00] |  |
|  |  | All-or-nothing | Coefficient | 0.12 | 0.44 | 1.24 | 0.06 | 1.29 | 4.00% |
|  |  |  | SE | 0.05 | 0.95 | 0.35 | 0.08 | 0.36 |  |
|  |  |  | p value | 0.015 | 0.642 | <0.001 | 0.489 | <0.001 |  |
|  |  |  | 95% CI | [0.02, 0.23] | [-1.42, 2.30] | [0.55, 1.93] | [-0.11, 0.22] | [0.58, 2.00] |  |
|  |  | Resting avoidance | Coefficient | 0.15 | 1.61 | 1.05 | 0.24 | 1.29 | 19.00% |
|  |  |  | SE | 0.05 | 1.30 | 0.35 | 0.15 | 0.36 |  |
|  |  |  | p value | 0.004 | 0.219 | 0.003 | 0.115 | <0.001 |  |
|  |  |  | 95% CI | [0.05, 0.25] | [-0.94, 4.16] | [0.36, 1.74] | [-0.06, 0.54] | [0.58, 2.00] |  |
| WPI | GAD7 | Fear avoidance | Coefficient | 0.19 | -0.69 | 1.00 | -0.13 | 0.88 | -15.00% |
|  |  |  | SE | 0.05 | 0.82 | 0.41 | 0.11 | 0.42 |  |
|  |  |  | p value | <0.001 | 0.402 | 0.015 | 0.254 | 0.036 |  |
|  |  |  | 95% CI | [0.09, 0.29] | [-2.30, 0.92] | [0.19, 1.81] | [-0.35, 0.09] | [0.06, 1.70] |  |
|  |  | Damage belief | Coefficient | 0.07 | 2.22 | 0.76 | 0.17 | 0.92 | 18.00% |
|  |  |  | SE | 0.04 | 1.24 | 0.41 | 0.11 | 0.42 |  |
|  |  |  | p value | 0.058 | 0.075 | 0.065 | 0.120 | 0.029 |  |
|  |  |  | 95% CI | [-0.002, 0.15] | [-0.21, 4.65] | [-0.04, 1.56] | [-0.04, 0.38] | [0.09, 1.75] |  |
|  |  | Symptom focusing | Coefficient | 0.17 | 1.79 | 0.53 | 0.30 | 0.83 | 36.00% |
|  |  |  | SE | 0.05 | 0.99 | 0.42 | 0.12 | 0.43 |  |
|  |  |  | p value | 0.001 | 0.071 | 0.209 | 0.012 | 0.054 |  |
|  |  |  | 95% CI | [0.07, 0.26] | [-0.15, 3.73] | [-0.29, 1.35] | [0.07, 0.53] | [-0.01, 1.67] |  |
|  |  | Embarrassment avoidance | Coefficient | 0.28 | 0.62 | 0.65 | 0.18 | 0.83 | 21.00% |
|  |  |  | SE | 0.07 | 0.92 | 0.42 | 0.10 | 0.43 |  |
|  |  |  | p value | <0.001 | 0.502 | 0.125 | 0.080 | 0.054 |  |
|  |  |  | 95% CI | [0.14, 0.42] | [-1.18, 2.42] | [-0.18, 1.48] | [-0.02, 0.38] | [-0.01, 1.67] |  |
|  |  | All-or-nothing | Coefficient | 0.11 | 1.50 | 0.67 | 0.16 | 0.84 | 19.00% |
|  |  |  | SE | 0.06 | 0.98 | 0.42 | 0.11 | 0.43 |  |
|  |  |  | p value | 0.065 | 0.126 | 0.114 | 0.135 | 0.050 |  |
|  |  |  | 95% CI | [-0.01, 0.23] | [-0.42, 3.42] | [-0.16, 1.50] | [-0.05, 0.37] | [0.001, 1.68] |  |
|  |  | Resting avoidance | Coefficient | 0.12 | 0.26 | 0.06 | 0.03 | 0.09 | 33.00% |
|  |  |  | SE | 0.06 | 0.15 | 0.05 | 0.02 | 0.05 |  |
|  |  |  | p value | 0.030 | 0.084 | 0.185 | 0.107 | 0.055 |  |
|  |  |  | 95% CI | [0.01, 0.23] | [-0.03, 0.55] | [-0.03, 0.15] | [-0.01, 0.07] | [-0.001, 0.18] |  |

| **Supplementary Table S11: Longitudinal mediating effect of CBRQ variables on PHQ9, GAD7 and Pain VAS (12-mo).** | | | | | | | | | |
| --- | --- | --- | --- | --- | --- | --- | --- | --- | --- |
| **Y** | **X** | **M** | **Estimates** | **a** | **b** | **c'** | **ab** | **c** | **Mediation** |
| Pain VAS | PHQ9 | Fear avoidance | Coefficient | 0.15 | 0.15 | 2.52 | 0.02 | 2.54 | 1.00% |
|  |  |  | SE | 0.04 | 1.28 | 0.46 | 0.19 | 0.43 |  |
|  |  |  | p value | <0.001 | 0.904 | <0.001 | 0.904 | <0.001 |  |
|  |  |  | 95% CI | [0.07, 0.23] | [-2.35, 2.66] | [1.63, 3.41] | [-0.35, 0.39] | [1.70, 3.39] |  |
|  |  | Damage belief | Coefficient | 0.08 | -1.32 | 2.66 | -0.11 | 2.55 | -4.00% |
|  |  |  | SE | 0.03 | 1.61 | 0.43 | 0.13 | 0.43 |  |
|  |  |  | p value | 0.012 | 0.413 | <0.001 | 0.417 | <0.001 |  |
|  |  |  | 95% CI | [0.02, 0.15] | [-4.48, 1.84] | [1.82, 3.50] | [-0.36, 0.15] | [1.71, 3.39] |  |
|  |  | Symptom focusing | Coefficient | 0.14 | 0.69 | 2.46 | 0.10 | 2.55 | 4.00% |
|  |  |  | SE | 0.04 | 1.40 | 0.43 | 0.21 | 0.43 |  |
|  |  |  | p value | 0.001 | 0.623 | <0.001 | 0.635 | <0.001 |  |
|  |  |  | 95% CI | [0.06, 0.23] | [-2.05, 3.43] | [1.62, 3.30] | [-0.31, 0.50] | [1.71, 3.40] |  |
|  |  | Embarrassment avoidance | Coefficient | 0.18 | 1.33 | 2.29 | 0.24 | 2.53 | 10.00% |
|  |  |  | SE | 0.05 | 1.17 | 0.43 | 0.14 | 0.43 |  |
|  |  |  | p value | <0.001 | 0.257 | <0.001 | 0.091 | <0.001 |  |
|  |  |  | 95% CI | [0.08, 0.28] | [-0.97, 3.63] | [1.45, 3.13] | [-0.04, 0.52] | [1.69, 3.37] |  |
|  |  | All-or-nothing | Coefficient | 0.17 | 1.32 | 2.32 | 0.23 | 2.55 | 9.00% |
|  |  |  | SE | 0.05 | 1.31 | 0.43 | 0.16 | 0.43 |  |
|  |  |  | p value | <0.001 | 0.316 | <0.001 | 0.142 | <0.001 |  |
|  |  |  | 95% CI | [0.08, 0.27] | [-1.25, 3.89] | [1.48, 3.16] | [-0.08, 0.54] | [1.71, 3.39] |  |
|  |  | Resting avoidance | Coefficient | 0.15 | 0.50 | 2.45 | 0.08 | 2.52 | 3.00% |
|  |  |  | SE | 0.05 | 1.53 | 0.43 | 0.24 | 0.43 |  |
|  |  |  | p value | 0.002 | 0.745 | <0.001 | 0.748 | <0.001 |  |
|  |  |  | 95% CI | [0.06, 0.25] | [-2.50, 3.50] | [1.61, 3.29] | [-0.39, 0.54] | [1.68, 3.36] |  |
| Pain VAS | GAD7 | Fear avoidance | Coefficient | 0.19 | 0.87 | 2.45 | 0.16 | 2.62 | 6.00% |
|  |  |  | SE | 0.04 | 1.37 | 0.57 | 0.26 | 0.54 |  |
|  |  |  | p value | <0.001 | 0.526 | <0.001 | 0.542 | <0.001 |  |
|  |  |  | 95% CI | [0.11, 0.28] | [-1.82, 3.56] | [1.33, 3.57] | [-0.35, 0.67] | [1.56, 3.68] |  |
|  |  | Damage belief | Coefficient | 0.15 | -1.65 | 2.89 | -0.25 | 2.63 | -10.00% |
|  |  |  | SE | 0.04 | 1.72 | 0.54 | 0.26 | 0.54 |  |
|  |  |  | p value | <0.001 | 0.339 | <0.001 | 0.348 | <0.001 |  |
|  |  |  | 95% CI | [0.07, 0.23] | [-5.02, 1.72] | [1.83, 3.95] | [-0.76, 0.26] | [1.57, 3.69] |  |
|  |  | Symptom focusing | Coefficient | 0.20 | 0.92 | 2.44 | 0.19 | 2.63 | 7.00% |
|  |  |  | SE | 0.05 | 1.50 | 0.54 | 0.31 | 0.54 |  |
|  |  |  | p value | <0.001 | 0.540 | <0.001 | 0.553 | <0.001 |  |
|  |  |  | 95% CI | [0.11, 0.30] | [-2.02, 3.86] | [1.38, 3.50] | [-0.42, 0.80] | [1.57, 3.69] |  |
|  |  | Embarrassment avoidance | Coefficient | 0.21 | 2.00 | 2.19 | 0.43 | 2.61 | 16.00% |
|  |  |  | SE | 0.06 | 1.25 | 0.55 | 0.18 | 0.54 |  |
|  |  |  | p value | <0.001 | 0.110 | <0.001 | 0.018 | <0.001 |  |
|  |  |  | 95% CI | [0.10, 0.33] | [-0.45, 4.45] | [1.11, 3.27] | [0.07, 0.78] | [1.55, 3.67] |  |
|  |  | All-or-nothing | Coefficient | 0.13 | 2.19 | 2.35 | 0.28 | 2.63 | 11.00% |
|  |  |  | SE | 0.05 | 1.40 | 0.54 | 0.19 | 0.54 |  |
|  |  |  | p value | 0.009 | 0.119 | <0.001 | 0.143 | <0.001 |  |
|  |  |  | 95% CI | [0.03, 0.23] | [-0.56, 4.94] | [1.29, 3.41] | [-0.09, 0.65] | [1.57, 3.69] |  |
|  |  | Resting avoidance | Coefficient | 0.13 | 1.25 | 2.46 | 0.16 | 2.62 | 6.00% |
|  |  |  | SE | 0.05 | 1.64 | 0.54 | 0.22 | 0.54 |  |
|  |  |  | p value | 0.009 | 0.446 | <0.001 | 0.463 | <0.001 |  |
|  |  |  | 95% CI | [0.03, 0.23] | [-1.97, 4.47] | [1.40, 3.52] | [-0.27, 0.59] | [1.56, 3.68] |  |

| **Supplementary Table S12: Longitudinal mediating effect of CBRQ variables on PHQ9, GAD7 and WPI (12-mo).** | | | | | | | | | |
| --- | --- | --- | --- | --- | --- | --- | --- | --- | --- |
| **Y** | **X** | **M** | **Estimates** | **a** | **b** | **c'** | **ab** | **c** | **Mediation** |
| WPI | PHQ9 | Fear avoidance | Coefficient | 0.15 | 0.02 | 0.27 | 0.003 | 0.27 | 1.00% |
|  |  |  | SE | 0.04 | 0.14 | 0.05 | 0.02 | 0.05 |  |
|  |  |  | p value | <0.001 | 0.904 | <0.001 | 0.904 | <0.001 |  |
|  |  |  | 95% CI | [0.07, 0.23] | [-0.26, 0.29] | [0.18, 0.37] | [-0.04, 0.04] | [0.18, 0.37] |  |
|  |  | Damage belief | Coefficient | 0.08 | -0.15 | 0.29 | -0.01 | 0.27 | -4.00% |
|  |  |  | SE | 0.03 | 0.18 | 0.05 | 0.01 | 0.05 |  |
|  |  |  | p value | 0.012 | 0.413 | <0.001 | 0.417 | <0.001 |  |
|  |  |  | 95% CI | [0.02, 0.15] | [-0.50, 0.20] | [0.20, 0.38] | [-0.04, 0.02] | [0.18, 0.37] |  |
|  |  | Symptom focusing | Coefficient | 0.14 | 0.08 | 0.26 | 0.01 | 0.27 | 4.00% |
|  |  |  | SE | 0.04 | 0.15 | 0.05 | 0.02 | 0.05 |  |
|  |  |  | p value | 0.001 | 0.623 | <0.001 | 0.635 | <0.001 |  |
|  |  |  | 95% CI | [0.06, 0.23] | [-0.23, 0.38] | [0.18, 0.36] | [-0.03, 0.05] | [0.18, 0.37] |  |
|  |  | Embarrassment avoidance | Coefficient | 0.18 | 0.15 | 0.25 | 0.03 | 0.27 | 10.00% |
|  |  |  | SE | 0.05 | 0.13 | 0.05 | 0.02 | 0.05 |  |
|  |  |  | p value | <0.001 | 0.257 | <0.001 | 0.091 | <0.001 |  |
|  |  |  | 95% CI | [0.08, 0.28] | [-0.11, 0.40] | [0.16, 0.34] | [-0.004, 0.06] | [0.18, 0.37] |  |
|  |  | All-or-nothing | Coefficient | 0.17 | 0.15 | 0.25 | 0.02 | 0.27 | 9.00% |
|  |  |  | SE | 0.05 | 0.14 | 0.05 | 0.02 | 0.05 |  |
|  |  |  | p value | <0.001 | 0.316 | <0.001 | 0.142 | <0.001 |  |
|  |  |  | 95% CI | [0.08, 0.27] | [-0.14, 0.43] | [0.16, 0.34] | [-0.01, 0.06] | [0.18, 0.37] |  |
|  |  | Resting avoidance | Coefficient | 0.15 | 0.06 | 0.26 | 0.01 | 0.27 | 3.00% |
|  |  |  | SE | 0.05 | 0.17 | 0.05 | 0.03 | 0.05 |  |
|  |  |  | p value | 0.002 | 0.745 | <0.001 | 0.748 | <0.001 |  |
|  |  |  | 95% CI | [0.06, 0.25] | [-0.27, 0.38] | [0.17, 0.36] | [-0.04, 0.06] | [0.18, 0.37] |  |
| WPI | GAD7 | Fear avoidance | Coefficient | 0.19 | 0.10 | 0.29 | 0.02 | 0.31 | 6.00% |
|  |  |  | SE | 0.04 | 0.15 | 0.07 | 0.03 | 0.06 |  |
|  |  |  | p value | <0.001 | 0.526 | <0.001 | 0.542 | <0.001 |  |
|  |  |  | 95% CI | [0.11, 0.28] | [-0.20, 0.39] | [0.16, 0.42] | [-0.04, 0.08] | [0.18, 0.43] |  |
|  |  | Damage belief | Coefficient | 0.15 | -0.18 | 0.34 | -0.03 | 0.31 | -10.00% |
|  |  |  | SE | 0.04 | 0.19 | 0.06 | 0.03 | 0.06 |  |
|  |  |  | p value | <0.001 | 0.339 | <0.001 | 0.348 | <0.001 |  |
|  |  |  | 95% CI | [0.07, 0.23] | [-0.56, 0.19] | [0.22, 0.46] | [-0.09, 0.03] | [0.18, 0.43] |  |
|  |  | Symptom focusing | Coefficient | 0.20 | 0.10 | 0.29 | 0.02 | 0.31 | 7.00% |
|  |  |  | SE | 0.05 | 0.17 | 0.06 | 0.03 | 0.06 |  |
|  |  |  | p value | <0.001 | 0.540 | <0.001 | 0.553 | <0.001 |  |
|  |  |  | 95% CI | [0.11, 0.30] | [-0.22, 0.43] | [0.16, 0.41] | [-0.05, 0.09] | [0.18, 0.43] |  |
|  |  | Embarrassment avoidance | Coefficient | 0.21 | 0.22 | 0.26 | 0.05 | 0.31 | 16.00% |
|  |  |  | SE | 0.06 | 0.14 | 0.06 | 0.02 | 0.06 |  |
|  |  |  | p value | <0.001 | 0.110 | <0.001 | 0.018 | <0.001 |  |
|  |  |  | 95% CI | [0.10, 0.33] | [-0.05, 0.49] | [0.13, 0.38] | [0.01, 0.09] | [0.18, 0.43] |  |
|  |  | All-or-nothing | Coefficient | 0.13 | 0.24 | 0.28 | 0.03 | 0.31 | 11.00% |
|  |  |  | SE | 0.05 | 0.15 | 0.06 | 0.02 | 0.06 |  |
|  |  |  | p value | 0.009 | 0.119 | <0.001 | 0.143 | <0.001 |  |
|  |  |  | 95% CI | [0.03, 0.23] | [-0.06, 0.55] | [0.15, 0.40] | [-0.01, 0.07] | [0.18, 0.43] |  |
|  |  | Resting avoidance | Coefficient | 0.13 | 0.14 | 0.29 | 0.02 | 0.31 | 6.00% |
|  |  |  | SE | 0.05 | 0.18 | 0.06 | 0.02 | 0.06 |  |
|  |  |  | p value | 0.009 | 0.446 | <0.001 | 0.463 | <0.001 |  |
|  |  |  | 95% CI | [0.03, 0.23] | [-0.22, 0.50] | [0.16, 0.41] | [-0.03, 0.07] | [0.18, 0.43] |  |
